# Expression profile of genes encoding allatoregulatory neuropeptides in females of the spider *Parasteatoda tepidariorum* (Araneae, Theridiidae)

**DOI:** 10.1371/journal.pone.0222274

**Published:** 2019-09-10

**Authors:** Marta Katarzyna Sawadro, Agata Wanda Bednarek, Agnieszka Ewa Molenda, Agnieszka Izabela Babczyńska

**Affiliations:** Department of Animal Physiology and Ecotoxicology, University of Silesia in Katowice, Bankowa, Katowice, Poland; Oxford Brookes University, UNITED KINGDOM

## Abstract

Allatoregulatory neuropeptides are multifunctional proteins that take part in the synthesis and secretion of juvenile hormones. In insects, allatostatins are inhibitors of juvenile hormone biosynthesis in the *corpora allata* while allatotropins, act as stimulators. By quantitative real-time PCR, we analyzed the gene expression of allatostatin A (Pt*ASTA*), allatostatin B (Pt*ASTB*), allatostatin C (Pt*ASTC*), allatotropin (Pt*AT*) and their receptors (Pt*ASTA-R*, Pt*ASTB-R*, Pt*ASTC-R*, Pt*AT*-R) in various tissues in different age groups of female spiders. In the presented manuscript, the presence of allatostatin A, allatostatin C, and allatotropin are reported in females of the spider *P*. *tepidariorum*. The obtained results indicated substantial differences in gene expression levels for allatoregulatory neuropeptides and their receptors in the different tissues. Additionally, the gene expression levels also varied depending on the female age. Strong expression was observed coinciding with sexual maturation in the neuroendocrine and nervous system, and to a lower extent in the digestive tissues and ovaries. Reverse trends were observed for the expression of genes encoding the receptors of these neuropeptides. In conclusion, our study is the first hint that the site of synthesis and secretion is fulfilled by similar structures as observed in other arthropods. In addition, the results of the analysis of spider physiology give evidence that the general functions like regulation of the juvenile hormone synthesis, regulation of the digestive tract and ovaries action, control of vitellogenesis process by the neuropeptides seem to be conserved among arthropods and are the milestone to future functional studies.

## Introduction

Neuropeptides represent the largest class of signal compounds, which also include steroids and sesquiterpenoids. Based on protein sequence similarity and main physiological functions, insect neuropeptides are grouped into families. One of the largest neuropeptide family comprise the allatoregulatory neuropeptides [1, 2, 3].

Allatoregulatory neuropeptides are multifunctional proteins with conserved protein sequences [4]. They are commonly found in insects from which they were isolated for the first time. They have also been identified in various groups of arthropods as well as in non-arthropod groups such as annelids and mollusks: *Cancer borealis* [5], *Carcinus maenas* [6], *Triops newberryi* [7], *Penaeus monodon* [8], *Homarus americanus* [9], *Deroceras reticulatum* [10, 11, 12]. Numerous manuscripts report demonstrating allatoregulatory reactivity in basal metazoans [13]. In addition to conservation of protein sequence, this further indicates, that allatoregulatory signaling might be conserved among metazoans, and thus it might be an ancestral mechanism. Insects are often regarded as a reference group for studies of allatoregulatory peptides in other groups of arthropods due to in insects allatoregulatory neuropeptides have been described in most detail so far. Based on these studies in insects, one of the primary roles of allatoregulatory neuropeptides is the regulation of the juvenile hormones (JHs) biosynthesis. JHs in insects (and methyl farnesoate–a standard product in JH synthesis in insects, serve different functions in arthropods [14, 15]) control crucial processes such as molting, metamorphosis, ovary development, vitellogenesis, mating behavior, and oviposition. Recent studies also confirmed the role of JHs in morphogenesis and caste determination in social insects [16]. Therefore, allatoregulatory neuropeptides indirectly affect these JH-dependent processes.

Allatostatins (ASTs) have an inhibitory effect, whereas allatotropins (ATs) stimulate biosynthesis and release of the JHs by the *corpora allata* (CA) [2, 17, 18, 19, 20]. ASTs are a superfamily of invertebrate neuropeptides that were initially defined by their action as inhibitors of JH biosynthesis *in vivo*. To date, more than 60 types of ASTs have been isolated and characterized from a variety of insect species. These peptides can be classified into three groups: the FGL-allatostatin (A type), the W(X)6W allatostatin (B type), and the lepidopteran (*Manduca sexta*) allatostatin (C type) [19] while allatotropins are not divided into subgroups. They were first isolated from *M*. *sexta* (Manse-AT), and their sequence (GFKNVEMMTARGF-NH2) was confirmed based on cDNA and was characterized in other insects like *Spodoptera frugiperda* as well as *Aedes aegypti* [11].

Among arachnids, ASTs have been identified in ticks. A-type ASTs were detected in the synganglion of *Dermacentor variabilis* [21], whereas AST C (Manse-AST) was described in the central nervous system of *Ixodes scapularis* [22]. Despite the detection of these peptides in ticks, their role has not been revealed. Furthermore, there are only a few publications that are based on the immunohistochemical data, AST A protein localization in the central nervous system of the model spider species *Cupiennius salei* [23]. However, the quoted data do not provide information on the role of ASTs and AT in spiders (compare with [11]).

In insects, in which allatoregulatory neuropeptides are relatively well-known in comparison with other arthropods, neuropeptides are produced and secreted in the central nervous system by neurosecretory cells and interneurons [2, 17, 18, 19, 20]. The major neurosecretory systems of insects are located behind the central nervous system and include corpora cardiaca (CC) and CA glands together with their connections. The CC, are neurohaemal organs and are among the most essential structures of the neuroendocrine system of insects. They stores and release the neuropeptides which are synthesized in the neuroendocrine cells of the brain. This importance is further highlighted by the ability of the CC glands to produce certain peptides from specific neurosecretory cells themselves. Apart from the CC, CA glands are also responsible for releasing the neuropeptides in the neuroendocrine system in insects, but their primary function is the synthesis of the juvenile hormones [2].

The neuroendocrine system of insects regulates most critical metabolic, behavioral, homeostatic, developmental, and reproductive processes [1, 24], which are widely studied in spiders because they are among the most abundant invertebrate predators in terrestrial ecosystems, agroecosystems, and woodlands [25, 26, 27]. Moreover, spiders are crucial in the development of efficient, sustainable, low-input agricultural systems [28]. However, how such important spider behaviors as web building, molting, sexual maturation, and complex behaviors like parental care are controlled on the neurohormonal level is still insufficiently studied. Unfortunately, knowledge about the function of the neuroendocrine and nervous systems of spiders is still rudimentary. Many of the quoted publications refer to research conducted in the 1960s [29, 30, 31, 32, 33].

It seems that the neuroendocrine and nervous systems of spiders may be the site of neuropeptide synthesis. Bonaric [32] described the neuroendocrine complex (Schneider I organs + neurohemal organs) as the CC homolog and Schneider II organs as the structure homologous to insect CA. Loesel et al. [23] also confirmed that in addition to the neuroendocrine system in *C*. *salei*, other nervous neurosecretory cells might be responsible for the secretion of AST. In addition, the neurohemal organ (*tropfenkomplex*) was identified as another secretory organ, which stores substances produced in the central nervous system and transported through the Schneider I organ [32]. Since neuropeptides are neurotransmitters, it can be assumed that they are secreted into the hemolymph by exocytosis and are transported to target cells where they bind to specific receptors [34]. Therefore, to analyze the function of allatoregulatory neuropeptides, it is thus first necessary to identify their tissue of synthesis as well as receptor location.

The main aims of this study, which is the first attempt to investigate spiders allatoregulatory neuropeptides, were to (i) detect the presence of these substances in the model spider species *Parasteatoda tepidariorum* C. L. Koch, 1841 (Araneae, Theridiidae) and to (ii) identify the tissues where the synthesis takes place and target sites of their action by the determination of expression of genes encoding allatostatin A (AST A), allatostatin B (AST B), allatostatin C (AST C) and allatotropin (AT) as well as their receptors in various tissues of female spiders. In addition, age-dependent expression rates were investigated.

## Material and methods

### Spider breeding

*P*. *tepidariorum* females were obtained, from laboratory-bred strains of the Department of Animal Physiology and Ecotoxicology, University of Silesia. The animals were bred at 25 ± 1°C at 70% relative humidity under L: 16 h, D: 8 h photoperiodic cycle. They were fed with laboratory cultured *Drosophila melanogaster* or *D*. *hydei* and were irrigated regularly.

For the needs of the presented study, the following ontogenetic stages of *P*. *tepidariorum* females were selected: the penultimate nymphal stage (35th day of life), the last nymphal instar (38th day of life), mature females (40th day of life), females after mating (43rd day of life) and females after oviposition (47th day of life). The spider development time was counted from leaving the cocoon, according to Miyashita [35] and behavioral observations.

### Sample preparation

Five different tissues and organs of *P*. *tepidariorum*–the midgut glands with midgut (MG), neuroendocrine and nervous system (NS), ovaries (OV), hindgut (HG), and integument (INT)–were used for the analysis. Moreover, the entire body of adult spiders (EB) was used as a biological material. The number of tissues/organs per sample was determined during a pilot study and varied depending on the size and age of spiders (Table 1). The preliminary results were based on the analysis of the weight of tissues needed to obtain the proper concentration of total RNA, which was used to prepare a cDNA template in the reverse transcription process. The required weight of tissues/organs is correlated with the number of dissected individuals. Due to the efficiency of reverse transcription (80%), a minimum of 700 ng/μl of total RNA had to be isolated. For example, in the case of females in the penultimate nymphal stage (day 35 of life), due to the small size of the ovaries, it was necessary to dissect the ovaries from 20 spiders to obtain 700 ng/μl of total RNA (Table 1).

**Table 1:** The number of tissues/organs selected for detection of allatoregulatory neuropeptides in the spider *P*. *tepidariorum* depending on their stages of ontogenesis.

Tissues/organs	Stage of ontogenesis (day)				
	35th	38^th^	40th	43rd	47th
Neuroendocrine and nervous system	20	20	15	15	5
Ovaries	20	10	5	5	3
Integument	10	10	5	5	5
Midgut glands with midgut	10	5	5	5	3
Hindgut	20	20	15	15	5
Entire body	3	2	2	2	1

The tissues and organs were dissected on ice in sterile phosphate-buffered saline (PBS, 137 mM NaCl, 10 mM phosphate buffer (K_2_HPO_4_, KH_2_PO_4_), 2.7 mM KCl, pH 7.4). All tissues and organs were immediately frozen in liquid nitrogen and stored at -70°C until use. All samples were performed in six replicates.

### *In silico* search

AST A, AST B, AST C, and AT and their receptor protein sequences of different insects (see S1 Table for species and accession number from the NCBI protein database [36]) were used to search homologous proteins in *P*. *tepidariorum*. The IAAA00000000.1 (TSA 25-JUL-2015) version of the current Transcriptome Shotgun Assembly database was searched with default parameters of tblastn [37] and the candidates with based on the sequence homology criterion that was proposed by Pearson [38] were identified.

The transcript sequences that were selected this way have been searched for the presence of ORFs and then transcribed into protein sequences using the Geneious (ver. 9.1.2) software. These sequences were used to confirm the identity of the putative neuropeptides and neuropeptide receptor genes in *P*. *tepidariorum* with gene sequences of other arthropods. For this purpose, the Geneious, Clustal Omega and InterProScan [39] software and algorithms were used. Primers for the further analysis of the section of putative genes encoding the neuropeptides and neuropeptide receptors in *P*. *tepidariorum* were designed using the Geneious software (Table 2).

**Table 2:** Forward and reverse sequences for the Pt*ASTA*, Pt*ASTA-R*, Pt*ASTB*, Pt*ASTB-R*, Pt*ASTC*, Pt*ASTC-R*, Pt*AT*, Pt*AT-R* and Pt*RP49* primers.

Target genes	Forward primer sequence	Reverse primer sequence	Product size (bp)
Pt*ASTA*	5′-GAAAGCGAGACCTGAAGATAG-3′	5′-TGGTGCTCGTTGCCTAAT-3′	134
Pt*ASTA-R*	5′-CGATCGTTGGTATGCCATTG-3′	5′-GTATCGAGACTATGGCTTCTG-3′	129
Pt*ASTB*	5′-CCCTTCTCTTGCATCCATACT-3′	5′-GGCGATCACTGGTCATTTA-3′	134
Pt*ASTB-R*	5′-ACTATTTCAGGAGGTCATGTG-3′	5′-CTCGGTAGAACGGATAAGAAT-3′	100
Pt*ASTC*	5′-CAGTTGGATGGTTGATGATG-3′	5′-CGTTTCCTCTGAGGTCATT-3′	122
Pt*ASTC-R*	5′-ACTATTTCAGGGAGGCATGTG-3′	5′-CTCGGTAGCAACGGTAAGAAT-3′	100
Pt*AT*	5′-ATGTCGAATGCGCTGAT-3′	5′-CAACTTGATACATGGTGATTAAA-3′	109
Pt*AT-R*	5′-CCCTGGAGCCATCTATCTTAC-3′	5′-GCCACAGCCACAACAAA-3′	121
Pt*RP49*	5′-ACCAAGAGGTATTACAACAGAG-3′	5′-CTTGGATGATACAGCTGAGAG-3'	208

Possible gene duplication was checked by performing multiple sequence alignment of chosen sequences in Clustal Omega and their pairwise comparison in BLAST software. The criterion of duplicated gene proposed by Ouedraogo et al. [40] was used. Phylogenetic tree was constructed with MEGA X [41] using the Neighbor-Joining method [42] with a bootstrap of 2000 replicates [43] The tree is drawn to scale, with branch lengths in the same units as those of the evolutionary distances used to infer the phylogenetic tree (S1–S13 Figs)).

The bioinformatics analysis of the *P*. *tepidariorum* genome has indicated that some of the tested neuropeptides and their receptors have paralogs. Therefore, the data about gene duplication have not been placed in the manuscript and have been only used in the primer designing.

### RNA isolation and cDNA preparation

Total RNA was isolated from the tissues/organs using TRIzol Reagent (Invitrogen, Carlsbad, CA, USA) according to the standard method [44]. Remaining genomic DNA contamination was removed using the TURBO DNA-free kit (Ambion, Austin, TX, USA) according to the manufacturer’s protocol. Quality of RNA was assessed for using a NanoDrop 2000 spectrometer (Thermo Scientific, Wilmington, USA). Aliquots of 1 μg of total RNA were retrotranscripted using the Reverse Transcription System (Promega, Madison, WI, USA) and random primers according to the recommendations of the manufacturer. The reverse transcription products were then diluted with 80 μl of nuclear-free water. Prepared cDNA was used as the qPCR template.

### Quantitative real-time PCR (qPCR)

Relative expression of selected genes was quantified by real-time PCR performed in the LightCycler 480 System (Roche, Basel, Switzerland) using SYBR Green Select Master Mix (2x) (Applied Biosystems, Foster City, CA, USA). Reactions for each of the six biological replicates in all age groups were run in triplicates in a 15 μl volume. The reaction master mixes were prepared using SYBR Green Master Mix 2x (7.5 μl), 20 mM forward and reverse primer (0.48 μl), cDNA template (3 μl), and nuclear-free water (4.02 μl) in 96-well white plates (Roche, Basel, Switzerland). All qPCR reactions were performed under the following conditions: 3 minutes for the pre-PCR denaturation and polymerase activation step at 95°C, followed by 40 cycles of 15 s denaturation at 95°C, a 20 s hybridization step at 57°C and a 45 s elongation step at 72°C. The quantification and calculation of the gene expression levels were performed according to the standard curve method [45]. Standard curves for every gene of five dilution series (from 10^10^ to 10^6^ copies of DNA molecules) were constructed from purified cDNA (using the QIAquick PCR Purification Kit, following the manufacturer’s protocol). Melting curve analyses were performed to confirm the amplification of a single PCR product. The thermal profile for the melting curve determination began with incubation of 1 min at 60°C with a gradual increase in temperature (1°C/15 s) to 95°C, during which time changes in fluorescence were monitored. No-template control tubes, containing water instead of template mRNA, were run under the same conditions for each gene. Real-time PCR efficiencies were calculated using the standard curve methods and normalized to the expression of the housekeeping gene ribosomal protein 49 (Pt*RP49*) for each sample. Preliminary tests (not shown) of the most commonly used references genes (*RP49*, *RP18*, α-actin, β-actin, 3-phosphate dehydrogenase; [46]) revealed that only the Pt*RP49* gene was stably expressed in various tissues, regardless of the stage of spider ontogenesis.

### Statistics

The results are reported as mean values ± SD. Normality was checked using the Kolmogorov-Smirnov test. The data were tested for homogeneity of variance using Levene’s test of equality of error variances. Whenever a significant effect was observed, the Tukey multiple comparison test was used for a post hoc one-way analysis of variance (ANOVA). Analysis of variance for the level of genes encoding allatoregulatory neuropeptides (Pt*ASTA*, Pt*ASTB*, Pt*ASTC*, Pt*AT*) and their receptors (Pt*ASTA-R*, Pt*ASTB-R*, Pt*ASTC-R*, Pt*AT-R*) at different stages of ontogenesis of *P*. *tepidariorum* females was performed using two-way ANOVA with the experimental groups (various tissues and bands) and the stages of life as the sources of differences. Results with p≤ 0.05 were considered to be significant. The data were analyzed using GraphPad Prism ver. 6.

## Results and discussion

**Fig 1:**
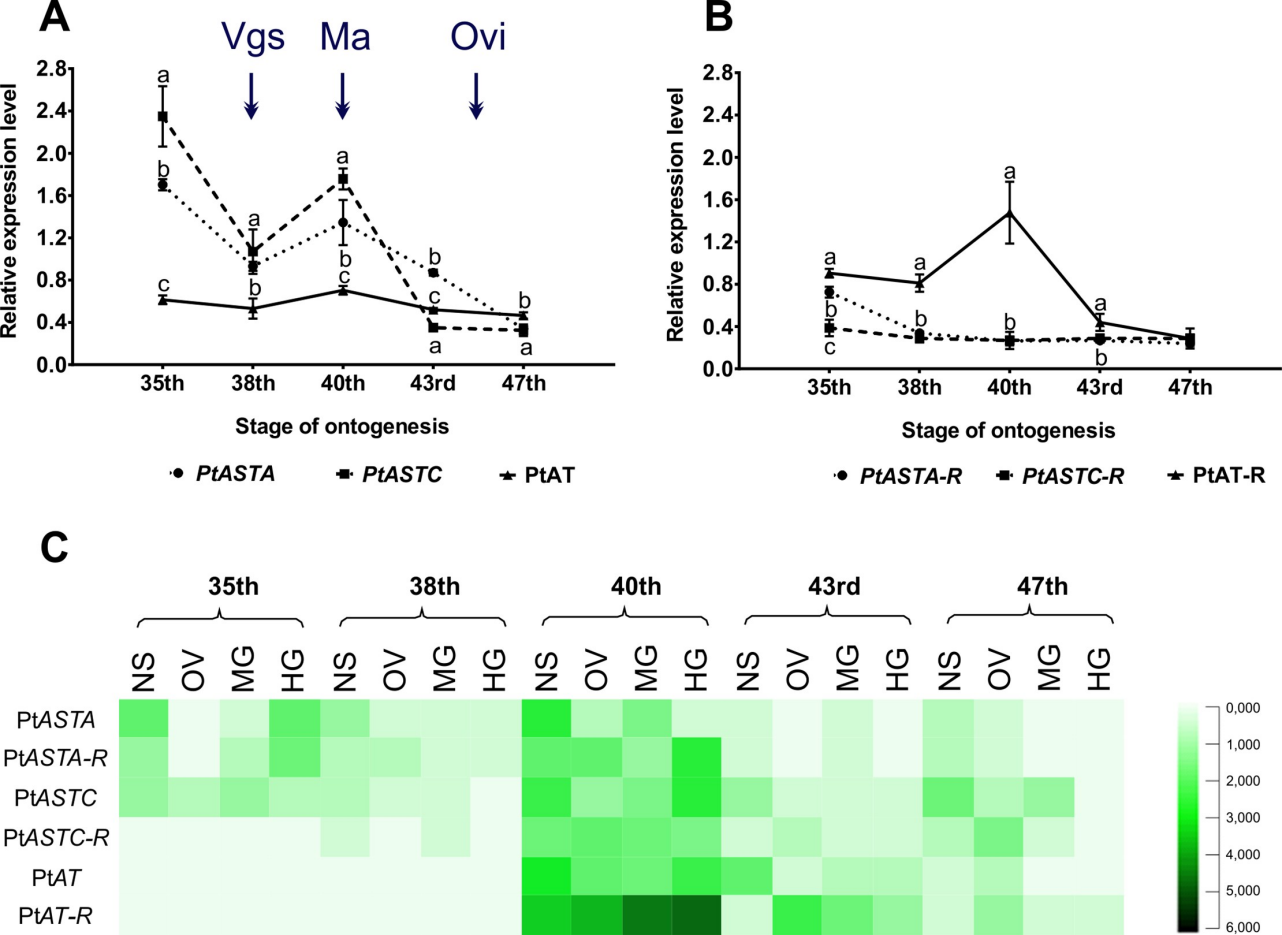
The relative expression level of genes encoding allatoregulatory neuropeptides in *Parasteatoda tepidariorum* females. Changes of the expression level of allatostatin A (Pt*ASTA*), allatostatin C (Pt*ASTC*), allatotropin (Pt*AT*) (A) and their receptors’ genes (Pt*ASTA-R*, Pt*ASTC-R*, Pt*AT-R*) (B) during the ontogenesis stages in the entire body of *P*. *tepidariorum* females. Differential expression of genes encoding allatoregulatory neuropeptides and their receptors in female (C) NS–neuroendocrine and nervous system, OV–ovaries, MG–midgut glands with midgut, HG–hindgut, Vgs–vitellogenesis process, Ma–mating, Ovi–oviposition.

Expression of genes encoding allatoregulatory neuropeptides and their receptors was visualized by a two-step real-time polymerase chain reaction (qPCR) method with cDNA as a template extracted from tissues/organs and from the entire body of *P*. *tepidariorum* females in various stages of ontogenesis. Proper products, corresponding to the expected fragments, were amplified for Pt*ASTA*, Pt*ASTC*, and Pt*AT* (Fig 1C). Among all tissues/ organs of the *P*. *tepidariorum* females analyzed in the experiment, no amplification was observed for genes encoding AST B and its receptor. The correct sequence of the allatostatin B receptor in the transcriptome of the *P*. *tepidariorum* spider has not been demonstrated by *in silico* analysis. The nucleotide sequences most closely related to ASTB-R do not contain TM1-TM7 conserved transmembrane domains (S7 Fig). It seems that AST B does not belong to the physiological neurosecretions of *P*. *tepidariorum*, and it does not play a role in the regulation of their physiological processes. On the other hand, this may also indicate a gene mutation resulting in a significant change of the nucleotide sequence in the genome. So far, among all allatostatins, peptides belonging to allatostatin B have been characterized and isolated only in five species of insects: *Locusta migratoria* [47], *M*. *sexta* [48], *Carausius morosus* [49], *Gryllus bimaculatus* [50] and *D*. *melanogaster* [51]. In contrast, allatostatin A and allatostatin C are widely distributed in various groups of insects. This may indicate a limited occurrence of this peptide in arthropods. Moreover, the potency of the B type allatostatins is approximately 50% lower than that of the A type allatostatins in *G*. *bimaculatus*. Lorenz et al. [52], as well as Wang [51], proved that the activity of AST B is limited to insects from the family of crickets (Gryllidae). These peptides isolated from *C*. *morosus* inhibited CA activity only in crickets but did not affect the CA of the *C*. *morosus* itself. Therefore, it can be assumed that limited occurrence of allatostatin B in insects and its limited activity may explain the possible lack of allatostatin B in *P*. *tepidariorum* females.

The obtained results confirmed the presence of allatoregulatory neuropeptides in *P*. *tepidariorum*. They revealed substantial differences in genes encoding allatoregulatory neuropeptides expression levels and their receptors in the different tissues, which may indicate the diverse production of neuropeptides and their subsequent action in the target tissues. The level of gene expression also varied depending on the age of individuals. However, for all transcripts excluding the entire body (Fig 1A and 1B), the highest expression level coincides with the beginning of sexual maturation at the 40th day of life.

It is clear that there might be more paralogs or that the genes observed are most likely homologs of the insect neuropeptides, but that a detailed phylogenetic analysis was not performed. It seems, that to confirm the presence of paralogs it would be necessary to perform phylogenetic analysis based on the newly available genome described by Posnien et al. [53] or Schwager et al. [54].

### Allatoregulatory neuropeptide expression in various tissues of mature females

**Fig 2:**
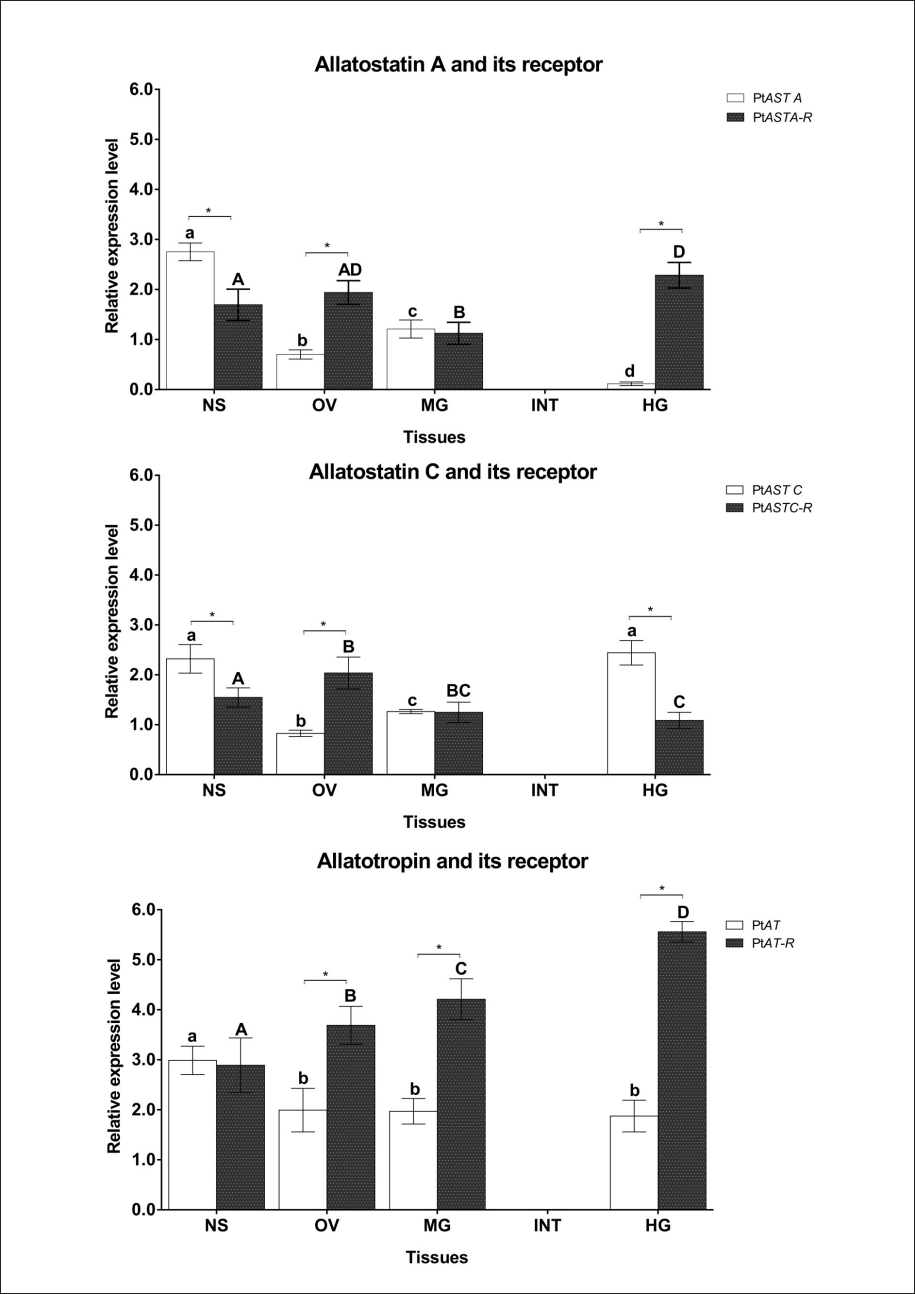
Tissue-specific expression of allatoregulatory neuropeptides. Relative expression of allatostatin A, allatostatin C, allatotropin and their receptors of *P*. *tepidariorum* females at the 40th day of ontogenesis (the day of sexual maturity) [mean ± SD]. NS–neuroendocrine and nervous system, OV–ovaries, MG–midgut glands with midgut, INT–integument, HG–hindgut (HG). Different letters indicate statistically significant differences: a-d differences between levels of expression of genes encoding allatoregulatory neuropeptides (Pt*ASTA*, Pt*ASTC*, Pt*AT*) in tested tissues, A-D differences between levels of expression of genes encoding allatoregulatory neuropeptide receptors (Pt*ASTA-R*, Pt*ASTC-R*, Pt*AT-R*) in tested tissues. Asterisks indicate statistically significant differences between levels of expression of gene encoding allatoregulatory neuropeptide and its receptor in the same tissue. Tukey's multiple comparison test, p≤0.05.

Among all tissues/organs of the female *P*. *tepidariorum* analyzed in the experiment, proper products of amplification for Pt*ASTA*, Pt*ASTC*, Pt*AT*, and their receptors were observed. The highest level of expression of AST A, AST C, and AT genes was recorded in the neuroendocrine and nervous systems. Moreover, the same level of expression for the gene encoding AST C was observed in the hindgut (Fig 2), whereas the maximum expression level of receptors was found in the hindgut for Pt*ASTA-R* and Pt*AT-R* as well as in the ovaries for Pt*ASTC-R* (Fig 2). In case of integument, no products of the amplification of all analyzed genes was obtained.

For all allatoregulatory neuropeptides significant differences in gene expression levels were found compared to their receptor expression. In the neuroendocrine and nervous systems, the relative level of Pt*ASTA* was significantly higher than the Pt*ASTA-R* level (Fig 2). The same correlation was observed for the gene encoding AST C and its receptor (Fig 2). In the remaining tissues, the inversed tendency was observed, where the expression of Pt*ASTA-R* gene was significantly higher than the expression of the gene encoding Pt*ASTA*. The most significant difference in the expression of the neuropeptide and its receptor was observed in the hindgut, where the expression of Pt*ASTA-R* was 20-fold higher than that of Pt*ASTA* (Fig 2). Analysis of the expression of Pt*ASTC* and Pt*ASTC-R* genes revealed that the relative level of Pt*ASTC* expression was also higher than the level of Pt*ASTC-R* in the hindgut. Furthermore, comparison of expression of the gene encoding the receptor of AT was higher than the level of Pt*AT* in all tissues/organs excluding neuroendocrine and nervous systems, where a significant difference between the level of both transcripts was not observed (Fig 2).

The highest expression of genes encoding Pt*ASTA*, Pt*ASTC*, and Pt*AT* in the neuroendocrine and nervous systems in comparison to other tissues of *P*. *tepidariorum* confirms the presence of neurosecretory cells, constituting the main site of synthesis and secretion of allatoregulatory neuropeptides. Our results are the first evidence consistently indicating the neuroendocrine and nervous system as a place of synthesis of allatoregulatory neuropeptides in spiders and confirming the assumptions described before by Bonaric [32]. Moreover, these results coincide with the data published for *C*. *salei*, where the presence of allatostatin A in the arcuate body of the central nervous system by immunochemistry method has been demonstrated [23]. This clearly confirmed the presence of allatostatins in the nervous system of spiders.

In addition, to the neuroendocrine and nervous systems, high differences of the transcripts levels have been demonstrated in the different tissues. The expression of genes encoding AST A, AST C, and AT was primarily confirmed in the ovaries, midgut glands with midgut and in the hindgut. In a study conducted by Stay et al. [55] in *Diploptera punctata* as well as by Witek and Hoffmann [56] in *Gryllus bimaculatus*, it was demonstrated that allatoregulatory neuropeptides can also be secreted by the ovaries, which would confirm the high expression of genes encoding these neuropeptides obtained in the ovaries.

The results of the expression of genes encoding the allatoregulatory neuropeptide receptors also indicate considerable variation according to tissue and ontogenesis stage. The expression of genes encoding the receptors of allatoregulatory neuropeptides was also demonstrated in the neuroendocrine and nervous systems, but its level was lower than in the other tissues. As explained earlier, the tissue referred to as the neuroendocrine and nervous systems contained all the structures of the neuroendocrine system described by Bonaric [29, 30, 31, 32] as well as by Bonaric and Juberthie [33]. In the aforementioned manuscripts, the neuroendocrine and nervous systems were selected as the site of JH synthesis and/or its analog as well as other substances belonging to the neurosecretions of spiders. The presence of allatoregulatory neuropeptide receptors in this complex of structures indicates the allatoregulatory control of the functioning of the neuroendocrine and nervous systems. We can assume that these neuropeptides influence the secretion of other neurotransmitters through neurosecretory cells of the neuroendocrine and nervous systems [1, 20, 57, 58]. To date, the expression of genes encoding allatoregulatory neuropeptide receptors in the nervous system of insects has been reported in *A*. *aegypti* [59], *D*. *melanogaster* [60] or *Apis mellifera* [61]. Similar results obtained in insects and for *P*. *tepidariorum* suggest that the allatoregulatory neuropeptides in spiders may play the primary control function of JH secretion. Additionally, it has been previously shown that AST can affect visual information processing [62], learning and memory [61]. The multitude of nervous system functions makes it difficult to deduce, based only on the expression of the studied transcripts.

Analysis of the expression of Pt*ASTA-R* and Pt*AT-R* genes confirmed their highest level in the hindgut, while the maximum expression level of the gene encoding the AST C receptor (Pt*ASTC-R*) was observed in the ovaries. The strong expression of analyzed genes in the hindgut may indicate the use of these neuropeptides for the regulation of gastrointestinal function as well as in intestinal peristaltic contractions, as in insects [58, 63, 64, 65, 66, 67]. Furthermore, it has also been shown that allatoregulatory neuropeptides control the secretion of digestive enzymes and stimulate their activity [68, 69, 70]. It cannot be excluded that similar mechanisms may occur in spiders. This conclusion is confirmed by the high expression of these transcripts in the midgut glands with midgut responsible for the production and secretion of digestive enzymes in spiders. The obtained results may also indicate the participation of allatoregulatory neuropeptides in other processes that take place in the midgut glands and midgut: energy metabolism processes, protein synthesis, and mobilization of spare substances. Protein synthesis in midgut glands and the midgut is also associated with the initiation of the vitellogenesis process. Hitherto, there is only one publication reporting the effect of AST A on reducing the production of vitellogenins in the fat body, as well as the inhibition of secretion of these proteins to hemolymph in *B*. *germanica* [71].

Proper products, corresponding to the expected fragments, amplified for genes encoding receptors of allatoregulatory neuropeptides in the ovaries suggest that they may regulate oocyte development, vitellogenesis, and choriogenesis, which has been confirmed by Woodhead et al. [72] in *D*. *punctata*, and the biosynthesis of ecdysone in the ovaries presented by Lorenz [73] in *G*. *bimaculatus* and Lorenz et al. [74] in *Blaptica dubia*.

Results from this study may confirm that in *P*. *tepidariorum* females the digestive tract and ovaries are the main tissues under allatoregulatory control. The process of vitellogenesis in *P*. *tepidariorum* is controlled on various levels (the gene expression, protein synthesis, protein deposition in ovaries) by allatoregulatory neuropeptides, indicated by allatotropin and allatostatin injections [75]. The allatoregulatory neuropeptides regulate the spider *P*. *tepidariorum* metabolism by inducing changes in the concentration of glycogen, lipids, and proteins in the midgut glands with midgut [76].

### Age-dependent allatoregulatory neuropeptide expression

The expression profile of genes encoding allatoregulatory neuropeptides and their receptors was dependent on the stage of ontogenesis of *P*. *tepidariorum* females. Analysis of the entire body of *P*. *tepidariorum* females revealed that the expression of Pt*AT*, Pt*ASTA-R*, and Pt*ASTC-R* was stable in most cases, throughout their life, except the 35th day of ontogenesis when the Pt*ASTA-R* expression was significantly higher than the level in the other stages (Fig 1A and 1B). In females at the last nymphal stage (38th day of life) and 3 days after copulation (43rd day of life), the level of Pt*ASTA* and Pt*ASTC* expression was significantly lower compared to the earlier stages of ontogenesis. Compared to other genes encoding allatoregulatory neuropeptides, a lower level of expression for the Pt*AT* gene in the entire body of *P*. *tepidariorum* females was confirmed (Fig 1A).

Expression of all transcripts in selected tissues or organs was also age-dependent (Fig 1C). The same relation of changes in the expression level as in the entire body was confirmed. The maximum level of expression of every gene was observed on the day of sexual maturity (40th day of ontogenesis) in all tested tissues, except for the expression of Pt*ASTA* in the hindgut, where the highest expression level was observed in the penultimate nymphal stage. The highest expression of transcripts on sexual maturation day may indicate an increase in neuropeptide production. Such dependence may indicate the role of these substances in physiological processes that take place very intensively during that period. Among them, vitellogenesis is the essential process; it begins in spiders in the last nymphal stage [77] and intensifies significantly when sexual maturity is reached. Because vitellogenesis requires high energy expenditure, the high level of expression of transcripts in the midgut glands with midgut may also indicate their influence on the regulation of energy mobilization from the energy storage compounds.

Moreover, on the 38th and 43rd days of life, the relative expression levels of Pt*ASTA*, Pt*ASTA-R*, Pt*ASTC*, and Pt*ASTC-R* were lower than in the earlier stages of ontogenesis. In the neuroendocrine and nervous systems as well as in the midgut glands with midgut the expression levels of Pt*ASTA*, Pt*ASTC* and Pt*ASTA-R* transcripts on the 38th day were twice as low as on the 35th day, whereas in the ovaries the expression level of Pt*ASTC* was 11-fold lower. Inhibition of the expression of genes encoding ASTs at this stage of ontogenesis may indicate a reduction in the rate of physiological processes associated with the preparation of the body to achieve sexual maturity. The Pt*AT* gene expression pattern was different from genes encoding ASTs; Pt*AT* gene expression was not observed until the 40th day of individual development. The effect of AT on the secretion of JH and/or its analog, indispensable in development and larval growth, confirmed in insects may explain the lack of Pt*AT* gene expression on days 35 and 38 of ontogenesis. These results may confirm that the synthesis of AT in *P*. *tepidariorum* females does not occur in the nymphal stages. In insects, it has been proved that larval-larval molting and metamorphosis can only occur as a result of the significant reduction of the JH level, which inhibits both of these processes [78, 79]. Based on the main AT function in insects, which is the stimulation of JH secretion, it may be relatively easy to explain the lack of its expression in the nymphal stages. A high level of JH due to the presence of allatotropin would prevent the molting and growing of spiders. This is also confirmed by the high level of allatotropin after the last molt (the day of sexual maturity) and the level of gene expression encoding enzyme of the juvenile hormone biosynthetic pathway (Pt*CYP15A1* enzyme responsible for catalyzing the epoxidation of methyl farnesoate to the juvenile hormone) in the same ontogenesis stages of *P*. *tepidariorum*. Bednarek et al. [80] confirmed the lack of Pt*CYP15A1* transcript in nymphal stages and high level of this product on the day of sexual maturity.

It was also observed that on day 43 of life the expression in the neuroendocrine and nervous systems was 7-fold lower in the case of genes encoding AST A and nearly 6-fold lower for the AST A receptor gene in relation to the day of sexual maturity. The changes in Pt*ASTC* and Pt*ASTC-R* expression levels were similar, but the differences were much more extensive. On the 43rd day of ontogenesis, a more significant difference in the obtained results was confirmed. In the midgut glands with midgut and hindgut, the expression of Pt*ASTC* was 14-fold lower with respect to the results from the 40th day of ontogenesis, whereas expression of the Pt*ASTC-R* gene in the midgut glands with midgut at the 43rd day of development was 20-fold lower compared to the previous stage of ontogenesis. Again, higher levels of expression of the Pt*ASTA*, *PtASTA-R*, *PtASTC*, and Pt*ASTC-R* transcripts were confirmed in the neuroendocrine and nervous systems and the ovaries in females after the formation of the first cocoons (47th day). In other tissues, neither transcript was expressed during the day of ontogenesis. The overlap of results obtained for genes encoding the allatoregulatory neuropeptides and results for genes encoding their receptors confirm the increased use of AST A and AST C during egg laying. The high expression of the Pt*ASTA* and Pt*ASTC* genes in the neuroendocrine and nervous system confirms their increased synthesis, while the highest expression of Pt*ASTA-R* and Pt*ASTC-R* genes in ovaries indicate their role in the regulation of the ovaries’ action. Garside et al. [81] reported that in *D*. *punctata* the expression of AST A (Dipp-AST A) was deficient in the ovaries during the initiation of vitellogenesis (analogous results on the 38th day of spider ontogenesis) and increased until spermatophore deposition. Immediately after this process, the level of Dipp-AST A was lower, and after laying eggs, it increased rapidly. However, Woodhead et al. [72] reported that the level of AST in *D*. *punctata* ovaries was increasing from 3 days after pupation, while after spermatophore deposition, AST A content rapidly increased, with the maximum level on the day of egg laying. The authors noted that the rapid increase in the level of ASTs in the ovaries is correlated with the process of choriogenesis.

In contrast, the expression profiles for Pt*AT* and Pt*AT-R* genes were different. No expression in the nymphal stages (35th and 38th) was detected, although, after sexual maturity, expression rates were significantly higher than Pt*ASTA* and Pt*ASTC* (Fig 1C). In all tissues, Pt*AT* gene expression decreased gradually from days 40 to 47 of spider development. The increased expression of both the Pt*ASTA* and Pt*ASTA-R* genes in the nymphal stages and the confirmed AST A activity in relation to the synthesis of JH indirectly confirms the occurrence of JH and/or its analog in *P*. *tepidariorum*. The secretion of AST A in the nymphal stages and the lack of AT secretion may indicate inhibition of JH synthesis and/or its analog, which enabled the proper course of molting. Such mechanisms of action of allatoregulatory neuropeptides have been confirmed in insects [58, 78, 79].

To conclude, the results demonstrated in this paper confirm the presence of allatoregulatory neuropeptides (AST A, AST C, and AT) in females of the spider *P*. *tepidariorum*, which complements the current knowledge of both the physiology of spiders and the occurrence of allatoregulatory neuropeptides. Moreover, the neuroendocrine and nervous systems have been reported to be the main sites of synthesis of these compounds, whereas the digestive tract and ovaries were primarily affected by the allatoregulatory control. The highest levels of all tested neuropeptides were observed on the 40th day of females’ lives. This indicates increased production of these proteins and their increased use during the physiological processes that took place on that day, like vitellogenesis, oocyte development, or mating. These results are a starting point for future functional studies, which will advance our understanding of the role of neuropeptides in the regulation of spider physiology.

## Supporting information

S1 Fig **Neighbor-joining phylogenetic analysis of allatostatin A (A) and its receptor (B) homologs from different arthropod species based on amino acid sequences.** The ASTA of *Parasteatoda tepidariorum* (contig: comp1697_Seq0, IAAA01003828), *Neocaridina deticulata* (AIY69121.1 GI:728678671), *Dapnia pulex* (EFX87432.1 GI:321476471), *Blatella germanica* (PSN41608), *Plutella xylostella* (AJM76767.1 GI:756767227), *Drosophila melanogaster* (NP_524489, NP_001287511), *Spodoptera frugiperda* (CAD32496.1 GI:30141900), *Galleria mellonella* (XP_026761889.1), *Ornithodoros moubata* (JAW06703.1 GI:1202296532), the ASTA-R of *Bombyx mori* (NP_001037035), *Spodoptera littoralis* (ASO76367), *Drosophila hydei* (XP_023178668.1), *Dapnia pulex* (EFX75149.1 GI:321464139), *Varroa destructor* (XP_022649206.1), *Ixodes scapularis* (EEC00437), *Rhipicephalus pulchellus* (JAA56937), *Nephila clavipes* (PRD30488), *Parasteatoda tepidariorum* (contig: comp10476_seq1, IAAA01020812) were used to construct the tree. Bootstrap values (2000 replicates) are displayed by the nodes. Evolutionary analyses were conducted in MEGA X (Kumar et al., 2018). The genetic distance is drawn to scale.
(TIF)

S2 Fig **Neighbor-joining phylogenetic analysis of allatostatin B (A) and its receptor (B) homologs from different arthropod species based on amino acid sequences.** The ASTB of *Tribolium castaneum* (NP_001137202 XP_001809338), *Gryllus bimaculatus* (CAG28935), *Amphibalanus amphitrite* (AFK81930.1 GI:388894364), *Daphnia magna* (JAN91848, JAL16932), *Parasteatoda tepidariorum* (contig: comp9494_seq1, IAAA01019103), *Neocaridina denticulate* (AIY69130.1 GI:728678901), the ASTB-R of *Dapnia pulex* (EFX84318.1 GI:321473350), *Daphnia magna* (KZS13422), *Homarus americanus* (QCB19933), *Parasteatoda tepidariorum* (contig: comp30900_seq0) were used to construct the tree. Bootstrap values (2000 replicates) are displayed by the nodes. Evolutionary analyses were conducted in MEGA X (Kumar et al., 2018). The genetic distance is drawn to scale.
(TIF)

S3 Fig **Neighbor-joining phylogenetic analysis of allatostatin C (A) and its receptor (B) homologs from different arthropod species based on amino acid sequences.** The ASTC of *Bombyx mori* (BAG68396.1 GI:195946964), *Clostera anastomosis* (AEM44669.1 GI:343480114), *Drosophila melanogaster* (NP_523542.1 GI:17981755), *Varroa jacobsoni* (XP_022700594), *Daphnia magna* (KZS21307), *Parasteatoda tepidariorum* (contig: comp6938_seq0), the ASTC-R of *Drosophila melanogaster* (NP_649040.2 GI:45550648), *Drosophila erecta* (XP_001972840), *Daphnia pulex* (EFX72686.1 GI:321461656), *Centruroides sculpturatus* (XP_023221360), *Limulus polyphemus* (XP_022238562.1), *Varroa destructor* (XP_022653394), *Nephila clavipes* (PRD26743), *Parasteatoda tepidariorum* (contig: comp30900_seq1) were used to construct the tree. Bootstrap values (2000 replicates) are displayed by the nodes. Evolutionary analyses were conducted in MEGA X (Kumar et al., 2018). The genetic distance is drawn to scale.
(TIF)

S4 Fig **Neighbor-joining phylogenetic analysis of allatotropin (A) and its receptor (B) homologs from different arthropod species based on amino acid sequences.** The AT of *Parasteatoda tepidariorum* (contig: 242845), *Bombyx mori* (NP_001037303.1 GI:112983784), *Manduca sexta* (AAB08759.1 GI:1556473), *Helicoverpa armigera* (AAT92286.1 GI:51011906), *Spodoptera frugiperda* (CAD48594.1 GI:46367638), *Ixodes scapularis* (EEC06620.1 GI:215496980), the AT-R of *Platynereis dumerilii* (AKQ63076.1), *Schistocerca gregaria* (AEX08666.2 GI:672390488), *Manduca sexta* (ADX66344.1 GI:323433877), *Aedes aegypti* (AEN03789.1 GI:344310426), *Bombus terrestris* (XP_012174018.1 GI:808145589), *Parasteatoda tepidariorum* (contig 186775) were used to construct the tree. Bootstrap values (2000 replicates) are displayed by the nodes. Evolutionary analyses were conducted in MEGA X (Kumar et al., 2018). The genetic distance is drawn to scale.
(TIF)

S5 Fig **Multiple alignment of predicted mature *P*. *tepidariorum* allatostatin A (A), allatostatin B (B), allatostatin C (C), allatotropin (D).** Conserved amino acids are shown in blue (exact amino acid), orange (nearly similar) and white (no conservation).
(TIF)

S6 Fig Multiple alignment of allatostatin A receptor from *P. tepidariorum* and other arthropods. Deduced sequences of ASTA-R were obtained from *Bombyx mori* (NP_001037035), *Spodoptera littoralis* (ASO76367), *Drosophila hydei* (XP_023178668.1), *Dapnia pulex* (EFX75149.1 GI:321464139), *Varroa destructor* (XP_022649206.1), *Ixodes scapularis* (EEC00437), *Rhipicephalus pulchellus* (JAA56937), *Nephila clavipes* (PRD30488), *Parasteatoda tepidariorum* (contig: comp10476_seq1, IAAA01020812). The transmembrane domains (TM1-TM7) are indicated by boxes. (*) represent conserved motif residues, (:) conservation between groups of strongly similar properties with a score greater than .5 on the PAM 250 matrix, (.) conservation between groups of weakly similar properties with a score less than or equal to .5 on the PAM 250 matrix, (**+**) indicate amino acids that are characteristic of class A GPCRs, (**#)** represent cysteine residues for disulfide bridge (between TM2-TM3 and TM4–TM5) or palmitoylation (intracellular domain), (lar domain), ((between TM2 matrix genetic distance is drawn to and (ΔΔΔ) represent the highly conserved DRY motif of class A G protein-coupled receptors.
(TIF)

S7 Fig Multiple alignment of allatostatin B receptor from *P. tepidariorum* and other arthropods. Deduced sequences of ASTB-R were obtained from *Dapnia pulex* (EFX84318.1 GI:321473350), *Daphnia magna* (KZS13422), *Homarus americanus* (QCB19933), *Parasteatoda tepidariorum* (contig: comp30900_seq0). (*) represent conserved motif residues, (:) conservation between groups of strongly similar properties with a score greater than .5 on the PAM 250 matrix, (.) conservation between groups of weakly similar properties with a score less than or equal to .5 on the PAM 250 matrix.
(TIF)

S8 Fig Multiple alignment of allatostatin C receptor from *P. tepidariorum* and other arthropods. Deduced sequences of ASTC-R were obtained from *Drosophila melanogaster* (NP_649040.2 GI:45550648), *Drosophila erecta* (XP_001972840), *Daphnia pulex* (EFX72686.1 GI:321461656), *Centruroides sculpturatus* (XP_023221360), *Limulus polyphemus* (XP_022238562.1), *Varroa destructor* (XP_022653394), *Nephila clavipes* (PRD26743), *Parasteatoda tepidariorum* (contig: comp30900_seq1). The transmembrane domains (TM1-TM7) are indicated by boxes. (*) represent conserved motif residues, (:) conservation between groups of strongly similar properties with a score greater than .5 on the PAM 250 matrix, (.) conservation between groups of weakly similar properties with a score less than or equal to .5 on the PAM 250 matrix, (**+**) indicate amino acids that are characteristic of class A GPCRs, (**#)** represent cysteine residues for disulfide bridge (between TM2-TM3 and TM4–TM5) or palmitoylation (intracellular domain), (lar domcate conserved putative phosphorylation sites for PKA/C and (ΔΔΔ) represent the highly conserved DRY motif of class A G protein-coupled receptors.
(TIF)

S9 Fig Multiple alignment of allatotropin receptor from *P. tepidariorum* and other arthropods. Deduced sequences of AT-R were obtained from *Platynereis dumerilii* (AKQ63076.1), *Schistocerca gregaria* (AEX08666.2 GI:672390488), *Manduca sexta* (ADX66344.1 GI:323433877), *Aedes aegypti* (AEN03789.1 GI:344310426), *Bombus terrestris* (XP_012174018.1 GI:808145589), *Parasteatoda tepidariorum* (contig 186775). The transmembrane domains (TM1-TM7) are indicated by boxes. (*) represent conserved motif residues, (:) conservation between groups of strongly similar properties with a score greater than .5 on the PAM 250 matrix, (.) conservation between groups of weakly similar properties with a score less than or equal to .5 on the PAM 250 matrix, (**#)** represent cysteine residues for disulfide bridge (between TM2-TM3 and TM4–TM5) or palmitoylation (intracellular domain).
(TIF)

S10 Fig Multiple alignment of allatostatin A precursors from *P. tepidariorum* and other arthropods. Deduced sequences of ASTA were obtained from *Parasteatoda tepidariorum* (contig: comp1697_Seq0, IAAA01003828), *Amphibalanus amphitrie* (AFK81929.1 GI:388894362), *Drosophila melanogaster* (NP_524489, NP_001287511), *Plutella xylostella* (AJM76767.1 GI:756767227), *Neocaridina deticulata* (AIY69121.1 GI:728678671). Predicted mature neuropeptides are indicated by boxes. (*) represent conserved motif residues, (:) conservation between groups of strongly similar properties with a score greater than .5 on the PAM 250 matrix, (.) conservation between groups of weakly similar properties with a score less than or equal to .5 on the PAM 250 matrix.
(TIF)

S11 Fig Multiple alignment of allatostatin B precursors from *P. tepidariorum* and other arthropods. Deduced sequences of ASTB were obtained from *Tribolium castaneum* (NP_001137202 XP_001809338), *Gryllus bimaculatus* (CAG28935), *Amphibalanus amphitrite* (AFK81930.1 GI:388894364), *Daphnia magna* (JAN91848, JAL16932), *Parasteatoda tepidariorum* (contig: comp9494_seq1, IAAA01019103). Predicted mature neuropeptides are indicated by boxes. (*) represent conserved motif residues, (:) conservation between groups of strongly similar properties with a score greater than .5 on the PAM 250 matrix, (.) conservation between groups of weakly similar properties with a score less than or equal to .5 on the PAM 250 matrix
(TIF)

S12 Fig Multiple alignment of allatostatin C precursors from *P. tepidariorum* and other arthropods. Deduced sequences of ASTC were *Bombyx mori* (BAG68396.1 GI:195946964), *Clostera anastomosis* (AEM44669.1 GI:343480114), *Drosophila melanogaster* (NP_523542.1 GI:17981755), *Varroa jacobsoni* (XP_022700594), *Daphnia magna* (KZS21307), *Parasteatoda tepidariorum* (contig: comp6938_seq0). Predicted mature neuropeptides are indicated by boxes. (*) represent conserved motif residues, (:) conservation between groups of strongly similar properties with a score greater than .5 on the PAM 250 matrix, (.) conservation between groups of weakly similar properties with a score less than or equal to .5 on the PAM 250 matrix.
(TIF)

S13 Fig Multiple alignment of allatotropin precursors from *P. tepidariorum* and other arthropods. Deduced sequences of AT were *Parasteatoda tepidariorum* (contig: 242845), *Bombyx mori* (NP_001037303.1 GI:112983784), *Manduca sexta* (AAB08759.1 GI:1556473), *Helicoverpa armigera* (AAT92286.1 GI:51011906), *Spodoptera frugiperda* (CAD48594.1 GI:46367638), *Ixodes scapularis* (EEC06620.1 GI:215496980). Predicted mature neuropeptides are indicated by boxes. (*) represent conserved motif residues, (:) conservation between groups of strongly similar properties with a score greater than .5 on the PAM 250 matrix, (.) conservation between groups of weakly similar properties with a score less than or equal to .5 on the PAM 250 matrix.
(TIF)

S1 File The real-time PCR results. (PDF)

S1 Table Allatostatin A, allatostatin B, allatostatin C and allatotropin and their receptor protein sequences of different insects species and accession number from the NCBI protein database used to search homologous proteins in *P*. *tepidariorum*.
(PDF)

## Abbreviations

AST: allatostatin
AST A: allatostatin A
AST B: allatostatin B
AST C: allatostatin C
AT: allatotropin
CA: corpora allata
CC: corpora cardiaca
EB: entire body
HG: hindgut
INT: integument
JHs: juvenile hormones
MG: midgut glands with midgut
NS: neuroendocrine and nervous system
OV: ovaries
