# Supplementary figures and images for: Expression profile of genes encoding allatoregulatory neuropeptides in females of the spider Parasteatoda tepidariorum (Araneae, Theridiidae)
Source: PLoS One. 2019 Sep 10;14(9):e0222274. doi: 10.1371/journal.pone.0222274 (PMC6736302; doi:10.1371/journal.pone.0222274)

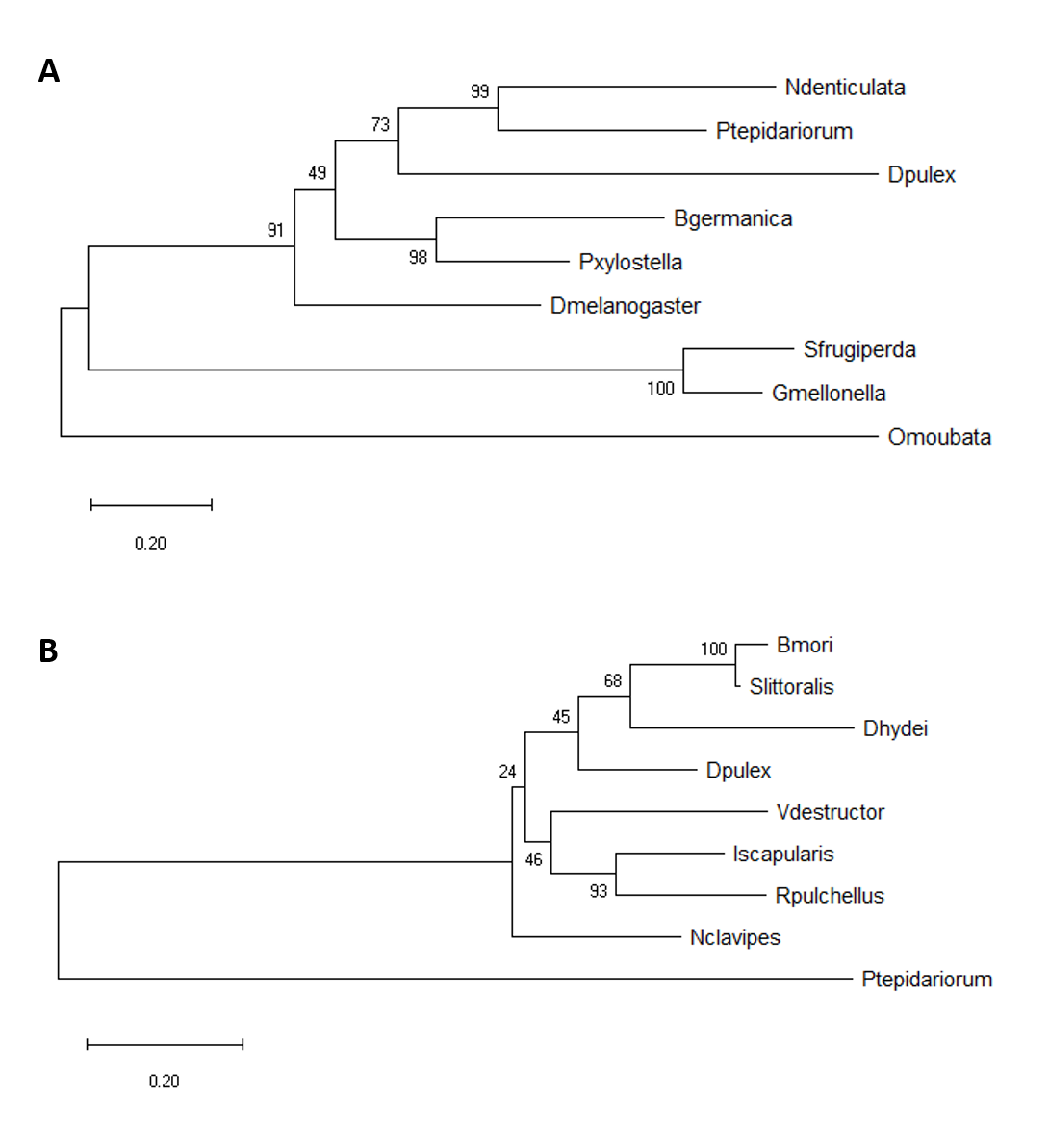

Supplement: S1 Fig — Neighbor-joining phylogenetic analysis of allatostatin A (A) and its receptor (B) homologs from different arthropod species based on amino acid sequences. The ASTA of Parasteatoda tepidariorum (contig: comp1697_Seq0, IAAA01003828), Neocaridina deticulata (AIY69121.1 GI:728678671), Dapnia pulex (EFX87432.1 GI:321476471), Blatella germanica (PSN41608), Plutella xylostella (AJM76767.1 GI:756767227), Drosophila melanogaster (NP_524489, NP_001287511), Spodoptera frugiperda (CAD32496.1 GI:30141900), Galleria mellonella (XP_026761889.1), Ornithodoros moubata (JAW06703.1 GI:1202296532), the ASTA-R of Bombyx mori (NP_001037035), Spodoptera littoralis (ASO76367), Drosophila hydei (XP_023178668.1), Dapnia pulex (EFX75149.1 GI:321464139), Varroa destructor (XP_022649206.1), Ixodes scapularis (EEC00437), Rhipicephalus pulchellus (JAA56937), Nephila clavipes (PRD30488), Parasteatoda tepidariorum (contig: comp10476_seq1, IAAA01020812) were used to construct the tree. Bootstrap values (2000 replicates) are displayed by the nodes. Evolutionary analyses were conducted in MEGA X (Kumar et al., 2018). The genetic distance is drawn to scale. (TIF) [file pone.0222274.s001.tif]

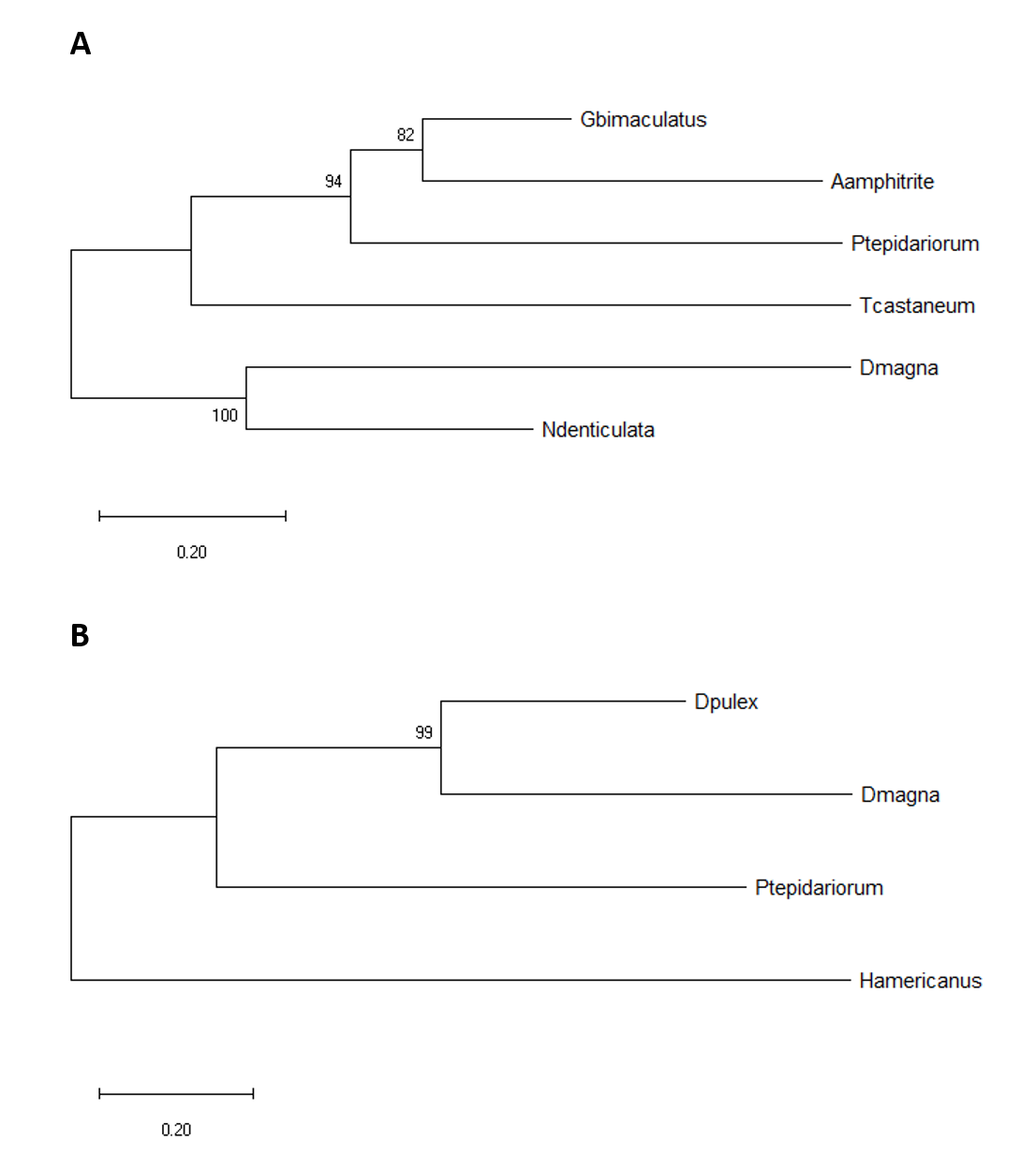

Supplement: S2 Fig — Neighbor-joining phylogenetic analysis of allatostatin B (A) and its receptor (B) homologs from different arthropod species based on amino acid sequences. The ASTB of Tribolium castaneum (NP_001137202 XP_001809338), Gryllus bimaculatus (CAG28935), Amphibalanus amphitrite (AFK81930.1 GI:388894364), Daphnia magna (JAN91848, JAL16932), Parasteatoda tepidariorum (contig: comp9494_seq1, IAAA01019103), Neocaridina denticulate (AIY69130.1 GI:728678901), the ASTB-R of Dapnia pulex (EFX84318.1 GI:321473350), Daphnia magna (KZS13422), Homarus americanus (QCB19933), Parasteatoda tepidariorum (contig: comp30900_seq0) were used to construct the tree. Bootstrap values (2000 replicates) are displayed by the nodes. Evolutionary analyses were conducted in MEGA X (Kumar et al., 2018). The genetic distance is drawn to scale. (TIF) [file pone.0222274.s002.tif]

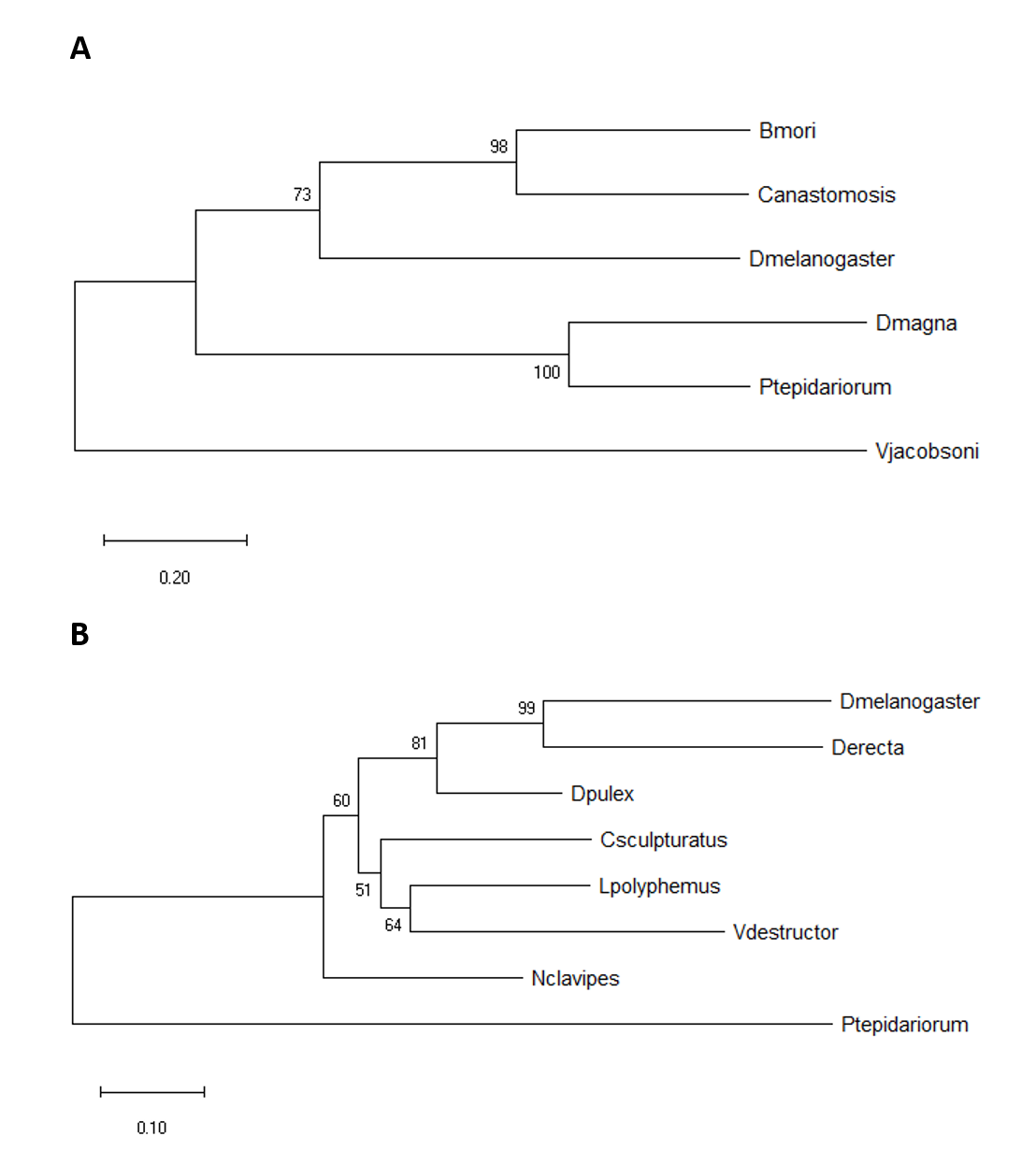

Supplement: S3 Fig — Neighbor-joining phylogenetic analysis of allatostatin C (A) and its receptor (B) homologs from different arthropod species based on amino acid sequences. The ASTC of Bombyx mori (BAG68396.1 GI:195946964), Clostera anastomosis (AEM44669.1 GI:343480114), Drosophila melanogaster (NP_523542.1 GI:17981755), Varroa jacobsoni (XP_022700594), Daphnia magna (KZS21307), Parasteatoda tepidariorum (contig: comp6938_seq0), the ASTC-R of Drosophila melanogaster (NP_649040.2 GI:45550648), Drosophila erecta (XP_001972840), Daphnia pulex (EFX72686.1 GI:321461656), Centruroides sculpturatus (XP_023221360), Limulus polyphemus (XP_022238562.1), Varroa destructor (XP_022653394), Nephila clavipes (PRD26743), Parasteatoda tepidariorum (contig: comp30900_seq1) were used to construct the tree. Bootstrap values (2000 replicates) are displayed by the nodes. Evolutionary analyses were conducted in MEGA X (Kumar et al., 2018). The genetic distance is drawn to scale. (TIF) [file pone.0222274.s003.tif]

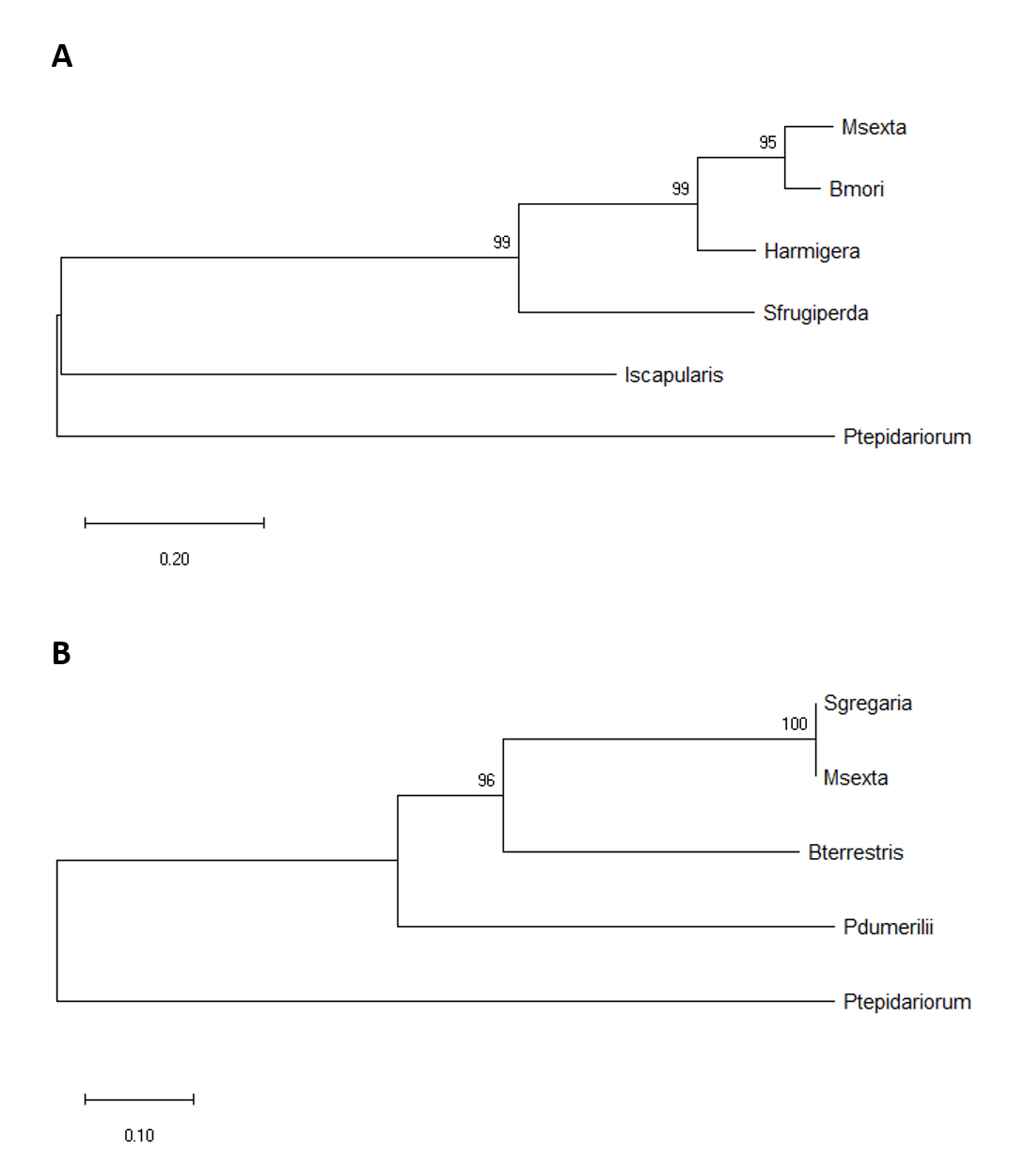

Supplement: S4 Fig — Neighbor-joining phylogenetic analysis of allatotropin (A) and its receptor (B) homologs from different arthropod species based on amino acid sequences. The AT of Parasteatoda tepidariorum (contig: 242845), Bombyx mori (NP_001037303.1 GI:112983784), Manduca sexta (AAB08759.1 GI:1556473), Helicoverpa armigera (AAT92286.1 GI:51011906), Spodoptera frugiperda (CAD48594.1 GI:46367638), Ixodes scapularis (EEC06620.1 GI:215496980), the AT-R of Platynereis dumerilii (AKQ63076.1), Schistocerca gregaria (AEX08666.2 GI:672390488), Manduca sexta (ADX66344.1 GI:323433877), Aedes aegypti (AEN03789.1 GI:344310426), Bombus terrestris (XP_012174018.1 GI:808145589), Parasteatoda tepidariorum (contig 186775) were used to construct the tree. Bootstrap values (2000 replicates) are displayed by the nodes. Evolutionary analyses were conducted in MEGA X (Kumar et al., 2018). The genetic distance is drawn to scale. (TIF) [file pone.0222274.s004.tif]

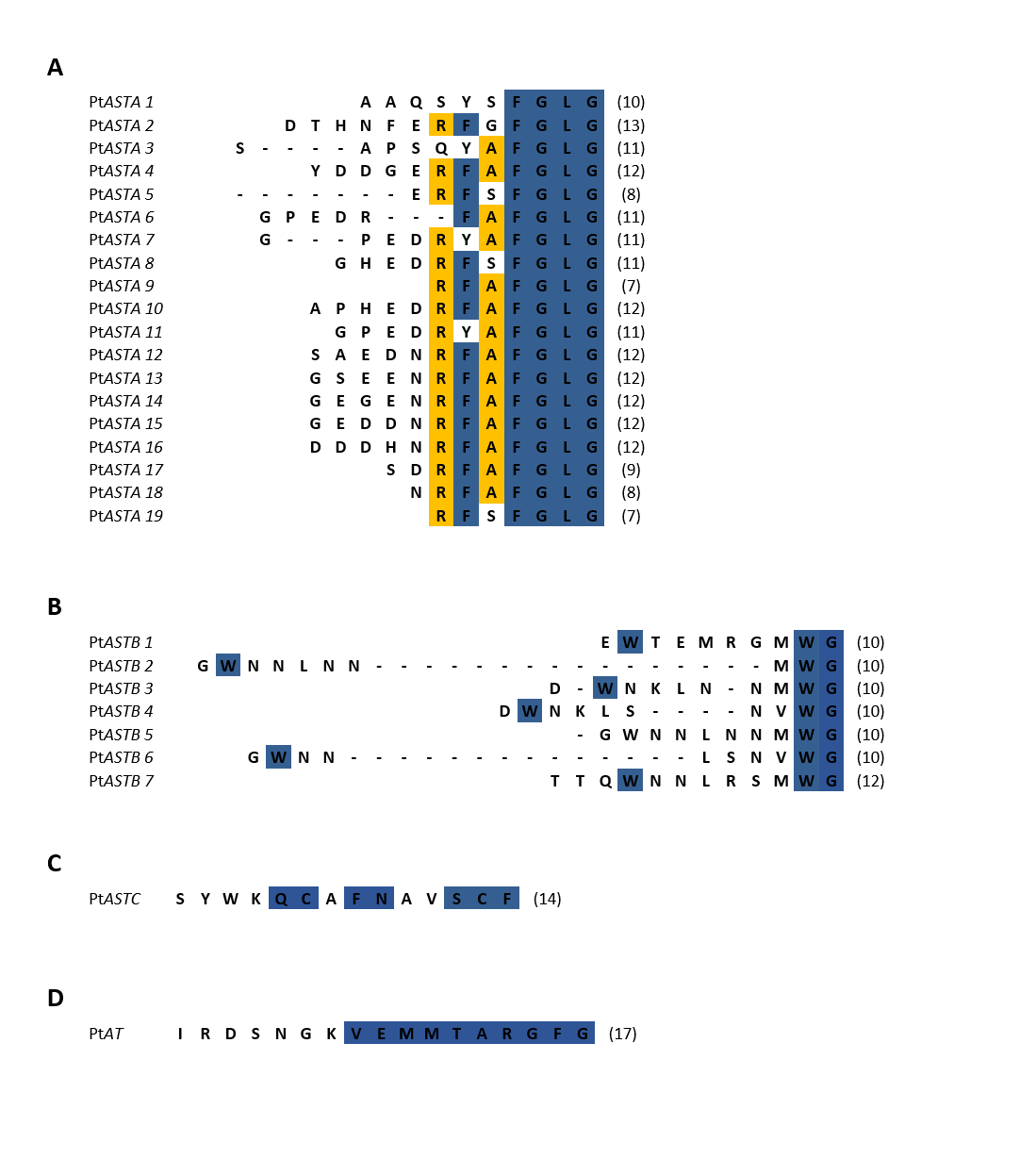

Supplement: S5 Fig — Multiple alignment of predicted mature P. tepidariorum allatostatin A (A), allatostatin B (B), allatostatin C (C), allatotropin (D). Conserved amino acids are shown in blue (exact amino acid), orange (nearly similar) and white (no conservation). (TIF) [file pone.0222274.s005.tif]

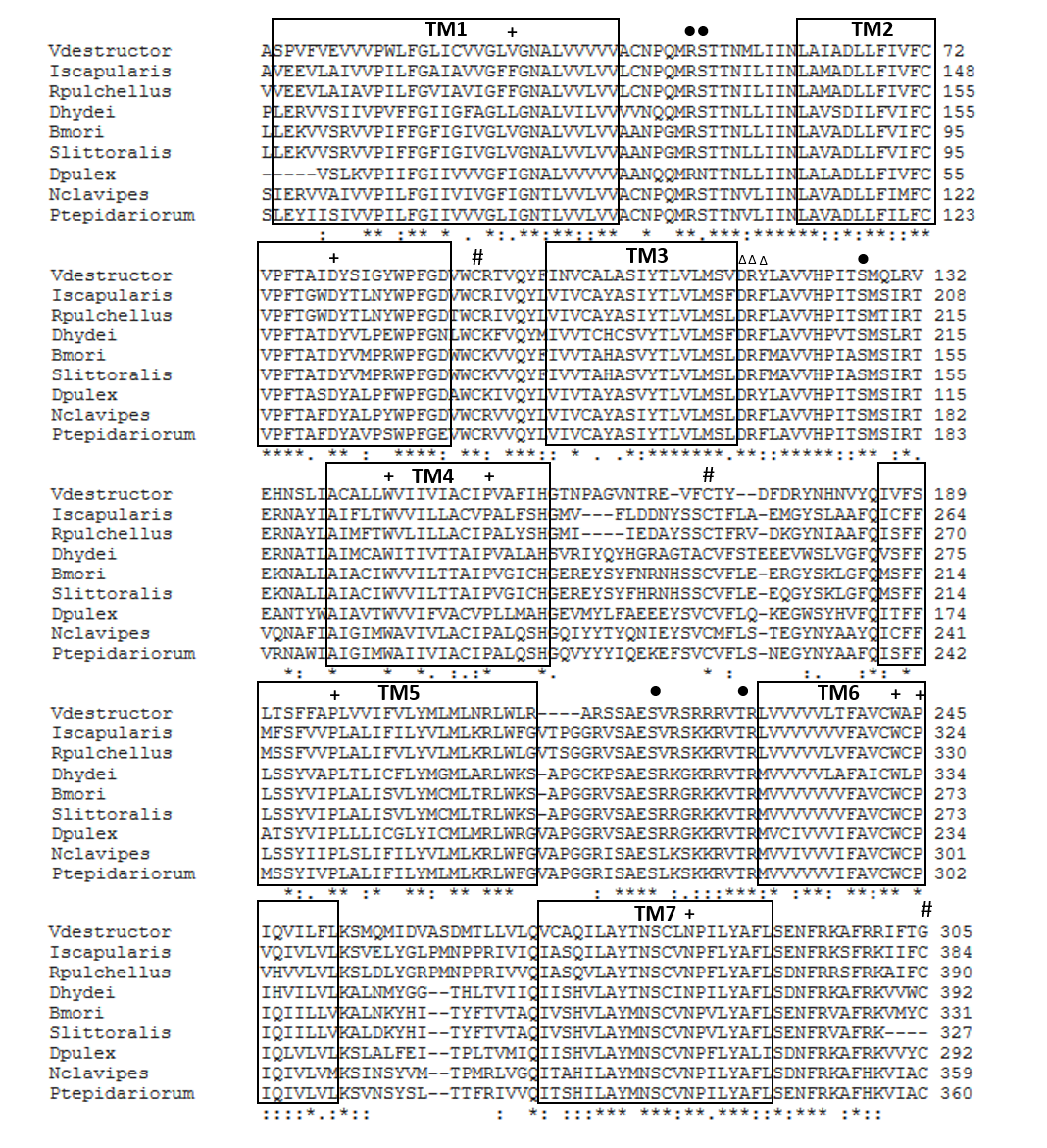

Supplement: S6 Fig — Deduced sequences of ASTA-R were obtained from Bombyx mori (NP_001037035), Spodoptera littoralis (ASO76367), Drosophila hydei (XP_023178668.1), Dapnia pulex (EFX75149.1 GI:321464139), Varroa destructor (XP_022649206.1), Ixodes scapularis (EEC00437), Rhipicephalus pulchellus (JAA56937), Nephila clavipes (PRD30488), Parasteatoda tepidariorum (contig: comp10476_seq1, IAAA01020812). The transmembrane domains (TM1-TM7) are indicated by boxes. (*) represent conserved motif residues, (:) conservation between groups of strongly similar properties with a score greater than .5 on the PAM 250 matrix, (.) conservation between groups of weakly similar properties with a score less than or equal to .5 on the PAM 250 matrix, (+) indicate amino acids that are characteristic of class A GPCRs, (#) represent cysteine residues for disulfide bridge (between TM2-TM3 and TM4–TM5) or palmitoylation (intracellular domain), (lar domain), ((between TM2 matrix genetic distance is drawn to and (ΔΔΔ) represent the highly conserved DRY motif of class A G protein-coupled receptors. (TIF) [file pone.0222274.s006.tif]

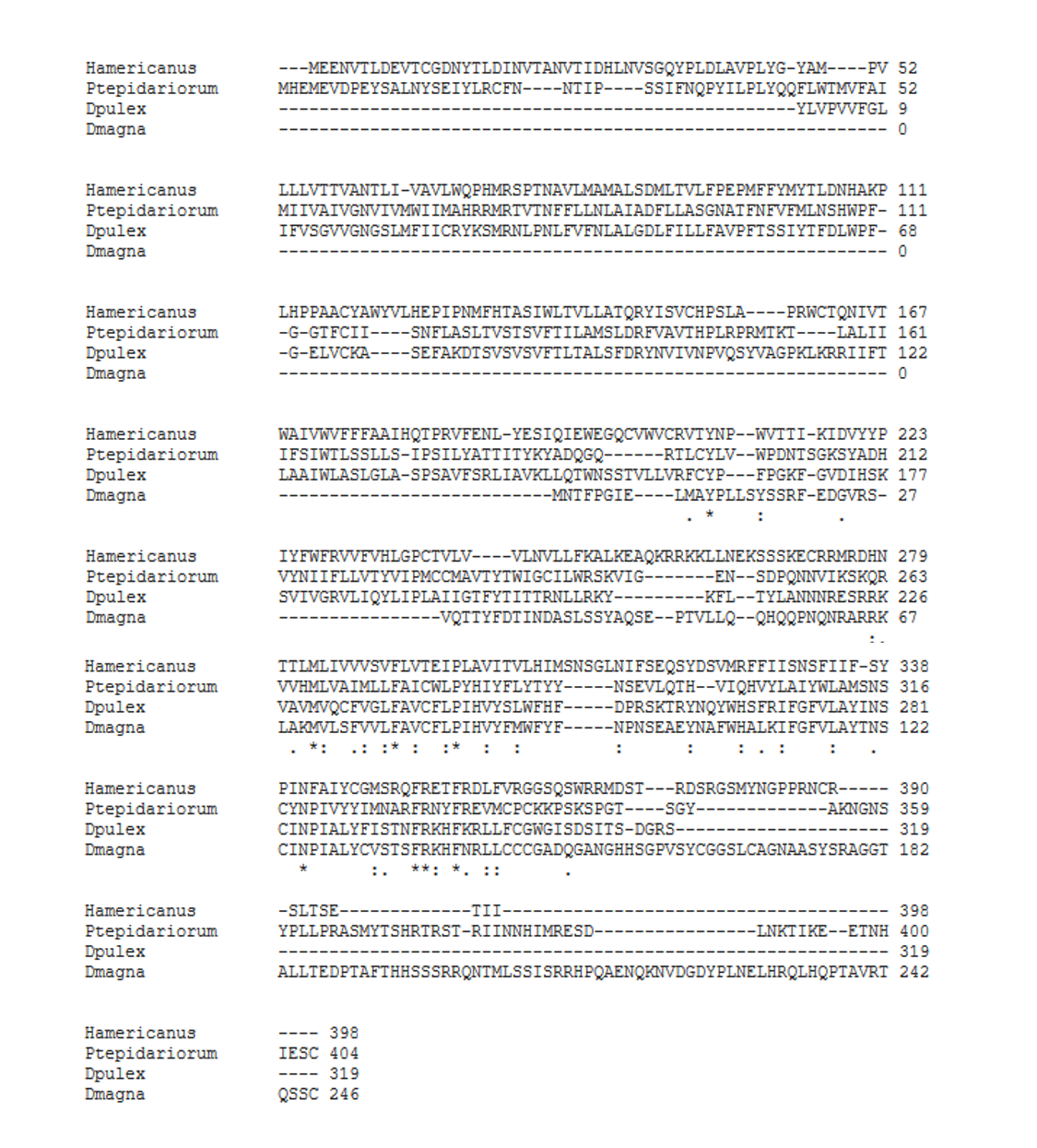

Supplement: S7 Fig — Deduced sequences of ASTB-R were obtained from Dapnia pulex (EFX84318.1 GI:321473350), Daphnia magna (KZS13422), Homarus americanus (QCB19933), Parasteatoda tepidariorum (contig: comp30900_seq0). (*) represent conserved motif residues, (:) conservation between groups of strongly similar properties with a score greater than .5 on the PAM 250 matrix, (.) conservation between groups of weakly similar properties with a score less than or equal to .5 on the PAM 250 matrix. (TIF) [file pone.0222274.s007.tif]

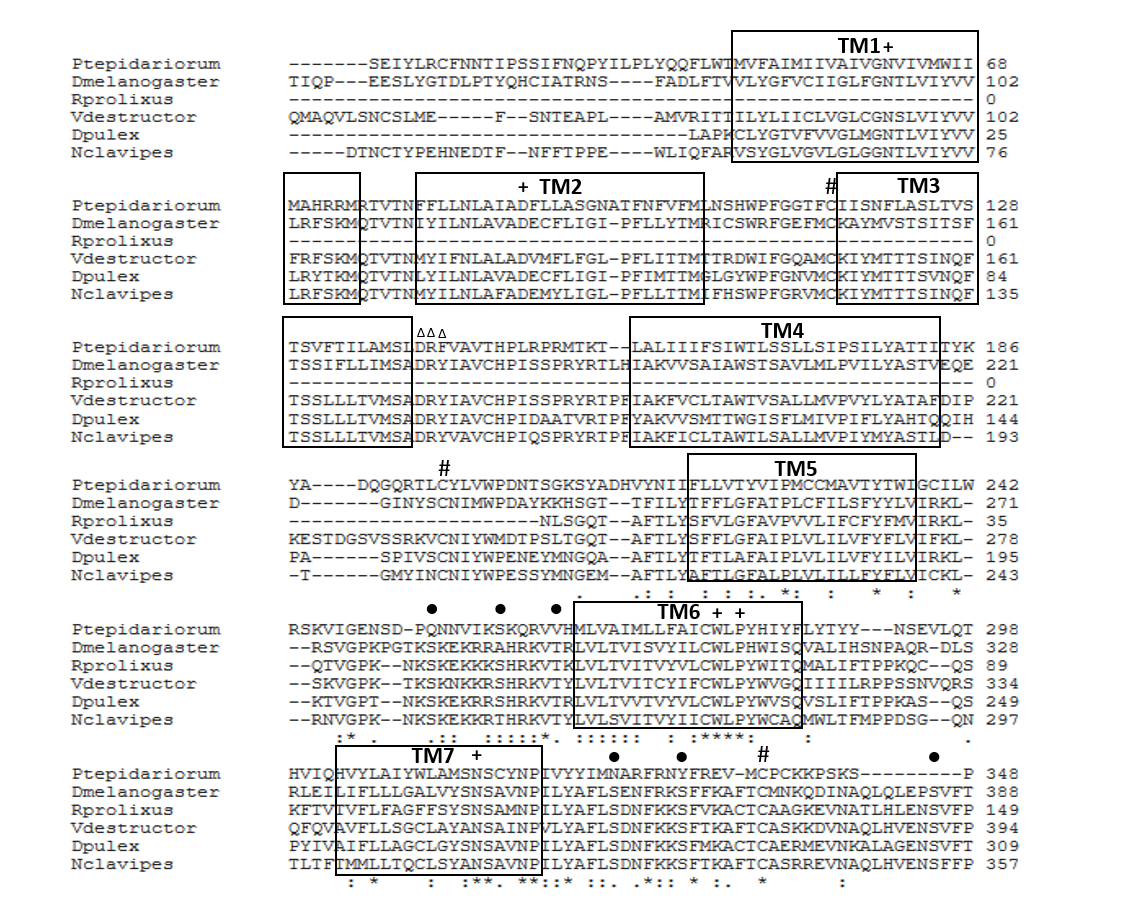

Supplement: S8 Fig — Deduced sequences of ASTC-R were obtained from Drosophila melanogaster (NP_649040.2 GI:45550648), Drosophila erecta (XP_001972840), Daphnia pulex (EFX72686.1 GI:321461656), Centruroides sculpturatus (XP_023221360), Limulus polyphemus (XP_022238562.1), Varroa destructor (XP_022653394), Nephila clavipes (PRD26743), Parasteatoda tepidariorum (contig: comp30900_seq1). The transmembrane domains (TM1-TM7) are indicated by boxes. (*) represent conserved motif residues, (:) conservation between groups of strongly similar properties with a score greater than .5 on the PAM 250 matrix, (.) conservation between groups of weakly similar properties with a score less than or equal to .5 on the PAM 250 matrix, (+) indicate amino acids that are characteristic of class A GPCRs, (#) represent cysteine residues for disulfide bridge (between TM2-TM3 and TM4–TM5) or palmitoylation (intracellular domain), (lar domcate conserved putative phosphorylation sites for PKA/C and (ΔΔΔ) represent the highly conserved DRY motif of class A G protein-coupled receptors. (TIF) [file pone.0222274.s008.tif]

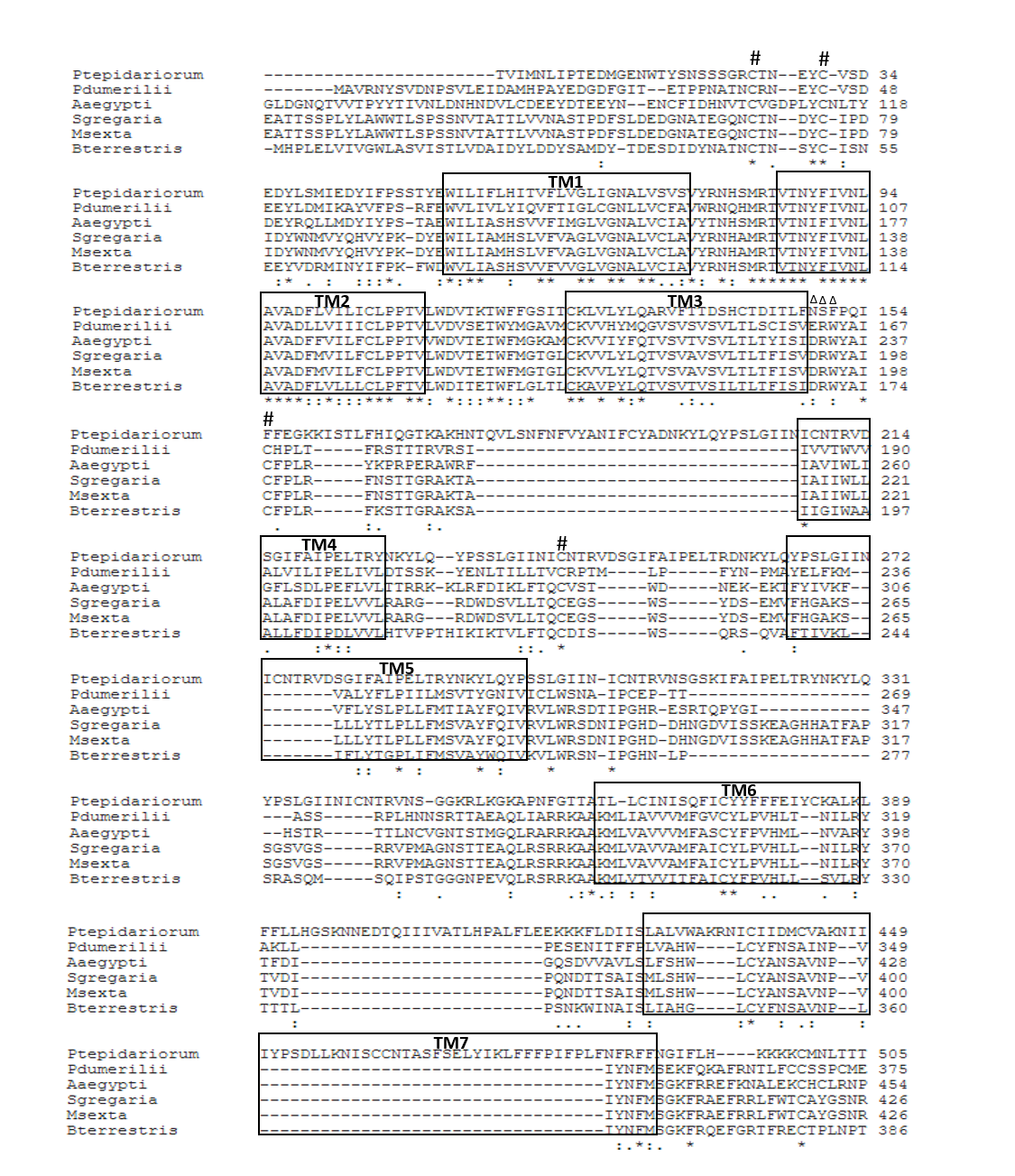

Supplement: S9 Fig — Deduced sequences of AT-R were obtained from Platynereis dumerilii (AKQ63076.1), Schistocerca gregaria (AEX08666.2 GI:672390488), Manduca sexta (ADX66344.1 GI:323433877), Aedes aegypti (AEN03789.1 GI:344310426), Bombus terrestris (XP_012174018.1 GI:808145589), Parasteatoda tepidariorum (contig 186775). The transmembrane domains (TM1-TM7) are indicated by boxes. (*) represent conserved motif residues, (:) conservation between groups of strongly similar properties with a score greater than .5 on the PAM 250 matrix, (.) conservation between groups of weakly similar properties with a score less than or equal to .5 on the PAM 250 matrix, (#) represent cysteine residues for disulfide bridge (between TM2-TM3 and TM4–TM5) or palmitoylation (intracellular domain). (TIF) [file pone.0222274.s009.tif]

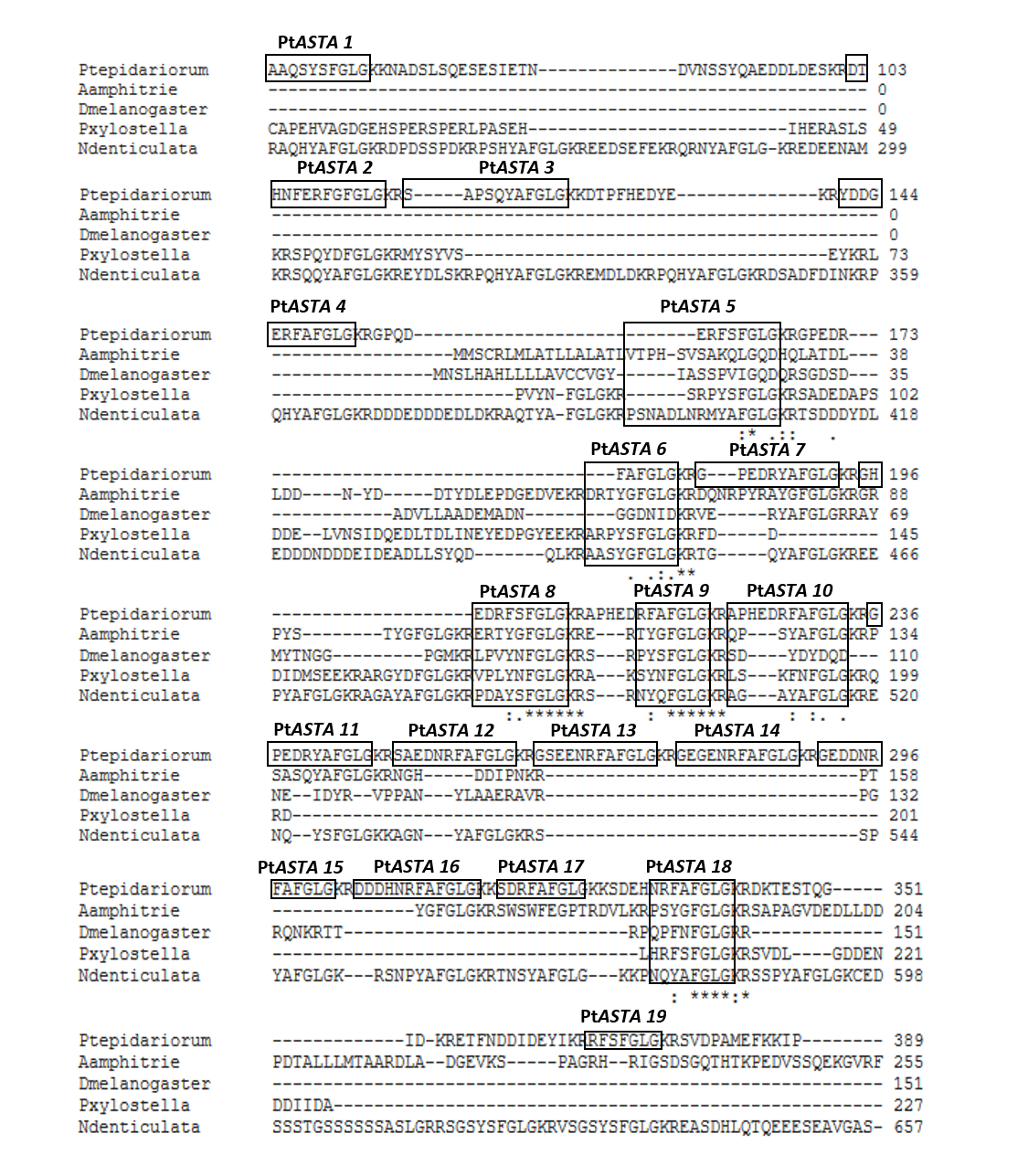

Supplement: S10 Fig — Deduced sequences of ASTA were obtained from Parasteatoda tepidariorum (contig: comp1697_Seq0, IAAA01003828), Amphibalanus amphitrie (AFK81929.1 GI:388894362), Drosophila melanogaster (NP_524489, NP_001287511), Plutella xylostella (AJM76767.1 GI:756767227), Neocaridina deticulata (AIY69121.1 GI:728678671). Predicted mature neuropeptides are indicated by boxes. (*) represent conserved motif residues, (:) conservation between groups of strongly similar properties with a score greater than .5 on the PAM 250 matrix, (.) conservation between groups of weakly similar properties with a score less than or equal to .5 on the PAM 250 matrix. (TIF) [file pone.0222274.s010.tif]

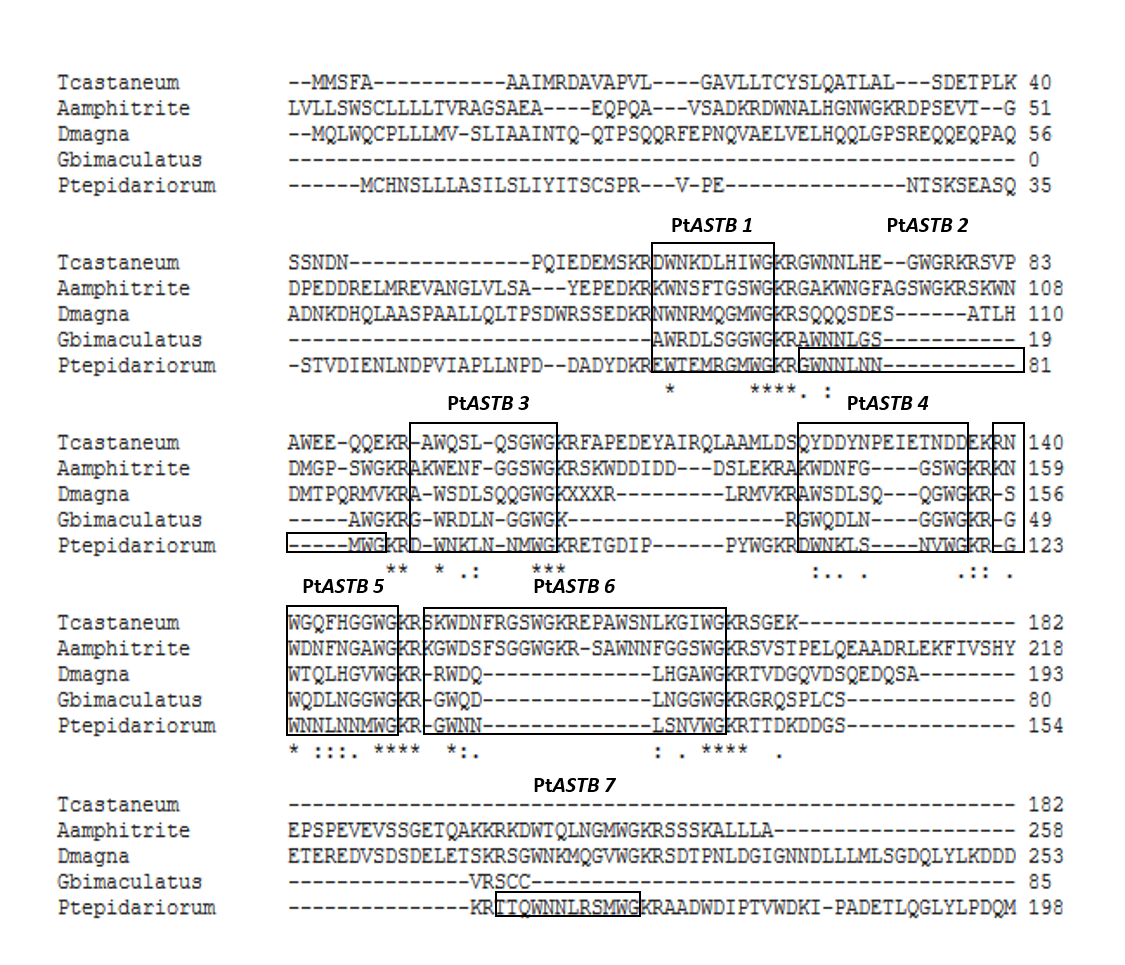

Supplement: S11 Fig — Deduced sequences of ASTB were obtained from Tribolium castaneum (NP_001137202 XP_001809338), Gryllus bimaculatus (CAG28935), Amphibalanus amphitrite (AFK81930.1 GI:388894364), Daphnia magna (JAN91848, JAL16932), Parasteatoda tepidariorum (contig: comp9494_seq1, IAAA01019103). Predicted mature neuropeptides are indicated by boxes. (*) represent conserved motif residues, (:) conservation between groups of strongly similar properties with a score greater than .5 on the PAM 250 matrix, (.) conservation between groups of weakly similar properties with a score less than or equal to .5 on the PAM 250 matrix (TIF) [file pone.0222274.s011.tif]

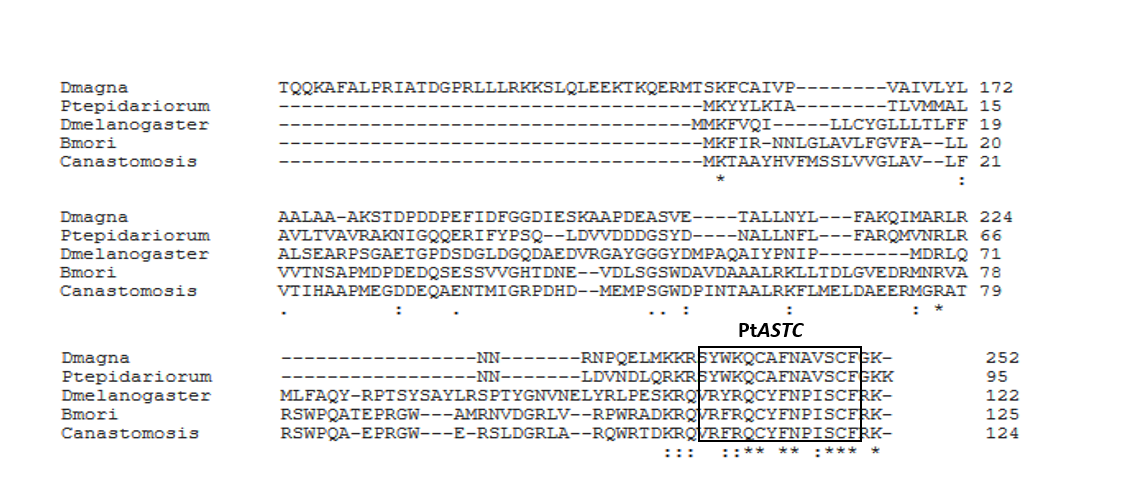

Supplement: S12 Fig — Deduced sequences of ASTC were Bombyx mori (BAG68396.1 GI:195946964), Clostera anastomosis (AEM44669.1 GI:343480114), Drosophila melanogaster (NP_523542.1 GI:17981755), Varroa jacobsoni (XP_022700594), Daphnia magna (KZS21307), Parasteatoda tepidariorum (contig: comp6938_seq0). Predicted mature neuropeptides are indicated by boxes. (*) represent conserved motif residues, (:) conservation between groups of strongly similar properties with a score greater than .5 on the PAM 250 matrix, (.) conservation between groups of weakly similar properties with a score less than or equal to .5 on the PAM 250 matrix. (TIF) [file pone.0222274.s012.tif]

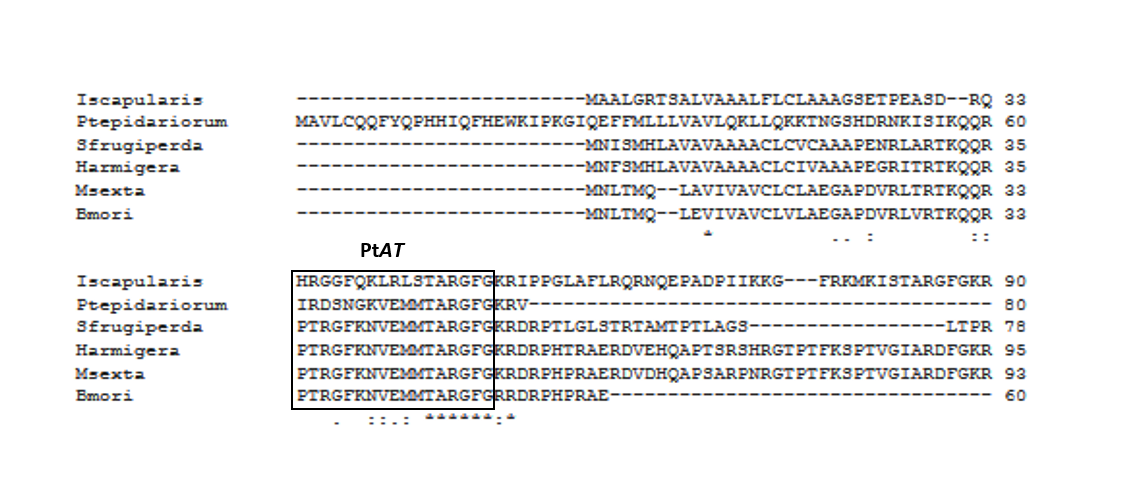

Supplement: S13 Fig — Deduced sequences of AT were Parasteatoda tepidariorum (contig: 242845), Bombyx mori (NP_001037303.1 GI:112983784), Manduca sexta (AAB08759.1 GI:1556473), Helicoverpa armigera (AAT92286.1 GI:51011906), Spodoptera frugiperda (CAD48594.1 GI:46367638), Ixodes scapularis (EEC06620.1 GI:215496980). Predicted mature neuropeptides are indicated by boxes. (*) represent conserved motif residues, (:) conservation between groups of strongly similar properties with a score greater than .5 on the PAM 250 matrix, (.) conservation between groups of weakly similar properties with a score less than or equal to .5 on the PAM 250 matrix. (TIF) [file pone.0222274.s013.tif]
