# Supplementary material for: Expression profile of genes encoding allatoregulatory neuropeptides in females of the spider Parasteatoda tepidariorum (Araneae, Theridiidae)
Source: PLoS One. 2019 Sep 10;14(9):e0222274. doi: 10.1371/journal.pone.0222274 (PMC6736302; doi:10.1371/journal.pone.0222274)
Supplement: S1 File — (PDF) [file pone.0222274.s014.pdf]

Gene	Cp									
	NS					OV				
	35th	38th	40th	43rd	47th	35th	38th	40th	43rd	47th
PtRP49	31,00	31,01	31,00	31,00	31,00	31,00	31,00	31,00	31,00	31,00
	31,00	31,00	31,00	31,00	31,00	31,00	31,00	31,00	31,00	31,00
	31,00	31,00	31,00	31,00	31,00	31,00	31,02	31,00	31,00	31,00
	31,00	31,00	31,00	31,00	31,00	31,00	31,00	31,00	31,00	31,00
	31,00	31,00	31,00	31,00	31,00	31,00	31,00	31,00	31,00	31,00
	31,00	31,00	31,00	31,00	31,00	31,00	31,00	31,00	31,00	31,00
PtASTA	17,43	28,41	7,12	34,67	30,03	0,00	35,35	30,76	0,00	36,33
	17,90	28,42	6,04	34,77	29,36	0,00	35,27	29,64	0,00	36,32
	19,59	28,42	9,84	33,20	30,12	0,00	35,59	31,44	0,00	36,34
	19,43	31,05	10,38	33,46	29,34	0,00	34,82	29,88	0,00	36,24
	17,90	28,51	9,94	34,67	30,03	0,00	35,27	30,97	0,00	34,93
	19,59	28,84	6,71	33,20	29,33	0,00	35,20	30,07	0,00	36,28
PtASTA-R	31,96	38,13	19,51	37,36	33,76	0,00	32,65	18,56	0,00	36,77
	32,45	38,13	19,40	38,55	36,26	0,00	32,78	18,58	0,00	38,04
	31,99	38,18	19,47	37,36	33,75	0,00	31,86	17,33	0,00	37,02
	31,99	36,88	28,16	38,58	35,84	0,00	34,86	11,29	0,00	37,90
	31,96	38,18	16,90	37,32	33,73	0,00	32,53	18,06	0,00	36,88
	31,96	31,64	19,14	38,59	35,76	0,00	32,64	18,58	0,00	38,01
PtASTC	28,65	33,75	16,17	30,23	24,03	31,98	37,54	29,58	37,16	34,40
	28,43	35,22	16,07	30,29	25,02	31,39	37,52	29,58	37,16	34,41
	28,44	34,22	16,13	30,26	24,12	32,39	37,66	29,61	37,16	34,16
	28,64	34,69	12,15	24,35	27,04	32,02	37,54	31,11	36,67	33,41
	28,43	34,19	17,13	29,37	24,02	32,09	37,52	29,90	37,10	34,24
	28,46	34,22	16,14	30,12	24,03	31,93	37,52	29,58	37,15	34,31
PtASTC-R	0,00	38,91	26,29	36,72	33,36	0,00	0,00	22,25	35,54	28,04
	0,00	38,86	26,29	36,91	33,34	0,00	0,00	15,07	33,83	29,05
	0,00	38,91	26,30	36,70	33,35	0,00	0,00	21,49	35,53	29,88
	0,00	38,86	26,18	36,73	33,38	0,00	0,00	22,31	35,54	28,17
	0,00	38,90	21,96	35,80	32,97	0,00	0,00	22,31	35,55	29,15
	0,00	38,86	26,29	36,26	33,36	0,00	0,00	22,24	34,26	28,13
PtAT	0,00	0,00	9,38	17,60	34,90	0,00	0,00	20,54	36,50	33,12
	0,00	0,00	9,32	17,52	34,88	0,00	0,00	20,54	36,51	33,12
	0,00	0,00	10,29	17,50	35,80	0,00	0,00	20,28	36,63	32,83
	0,00	0,00	15,62	11,20	34,90	0,00	0,00	11,54	36,50	31,41
	0,00	0,00	9,39	17,61	34,88	0,00	0,00	20,49	36,52	32,89
	0,00	0,00	9,36	17,50	34,89	0,00	0,00	20,52	36,60	33,10
PtAT-R	0,00	0,00	22,50	37,54	38,53	0,00	0,00	22,25	29,67	34,86
	0,00	0,00	27,47	37,68	38,77	0,00	0,00	19,80	29,65	34,85
	0,00	0,00	23,07	38,52	38,77	0,00	0,00	17,91	27,40	35,28
	0,00	0,00	22,59	37,54	38,53	0,00	0,00	19,46	32,11	36,33
	0,00	0,00	26,01	37,54	38,72	0,00	0,00	22,25	29,65	34,91
	0,00	0,00	27,42	37,54	38,77	0,00	0,00	22,23	29,65	34,89

Gene	Cp									
	MG					HG				
	35th	38th	40th	43rd	47th	35th	38th	40th	43rd	47th
PtRP49	31,00	31,00	31,00	31,00	31,00	31,00	31,00	31,00	31,00	31,00
	31,00	31,00	31,00	31,00	31,01	31,00	31,00	31,00	31,00	31,00
	31,00	31,00	31,00	31,00	31,00	31,00	31,00	31,00	31,00	31,00
	31,00	31,00	31,00	31,00	31,00	31,00	31,00	31,00	31,00	31,00
	31,01	31,00	31,00	31,00	31,00	31,00	31,00	31,00	31,00	31,00
	31,00	31,00	31,00	31,00	31,00	31,00	31,00	31,00	31,00	31,00
PtASTA	35,40	36,63	21,03	35,54	0,00	19,63	37,68	37,06	0,00	0,00
	35,05	35,99	25,94	35,99	0,00	26,40	37,88	36,08	0,00	0,00
	34,94	37,04	25,83	37,04	0,00	20,71	37,79	36,95	0,00	0,00
	35,17	36,81	25,83	35,54	0,00	19,73	37,69	37,05	0,00	0,00
	34,94	37,08	25,90	37,08	0,00	19,63	37,71	37,05	0,00	0,00
	35,17	35,97	25,83	35,52	0,00	19,71	37,68	37,05	0,00	0,00
PtASTA-R	34,33	37,90	28,42	38,06	0,00	23,98	37,32	11,54	0,00	0,00
	31,18	39,26	22,05	39,89	0,00	27,73	34,95	14,35	0,00	0,00
	34,21	38,70	29,67	39,01	0,00	23,97	37,33	14,23	0,00	0,00
	34,27	37,90	28,04	38,06	0,00	23,91	35,09	12,63	0,00	0,00
	34,31	39,26	27,17	39,89	0,00	23,93	36,83	14,24	0,00	0,00
	34,30	38,70	27,92	39,01	0,00	24,23	34,95	15,60	0,00	0,00
PtASTC	30,32	34,76	25,32	37,21	29,37	30,29	0,00	12,75	36,76	0,00
	30,32	34,80	26,06	37,19	29,57	30,89	0,00	13,89	34,89	0,00
	30,03	33,76	25,82	37,22	29,58	28,80	0,00	18,73	36,77	0,00
	30,63	34,79	26,32	37,11	29,58	30,79	0,00	12,97	36,68	0,00
	30,03	34,23	25,32	37,21	28,54	30,80	0,00	12,74	36,75	0,00
	30,63	34,76	25,33	37,20	29,55	30,79	0,00	12,95	36,73	0,00
PtASTC-R	0,00	38,12	29,36	39,26	37,59	0,00	0,00	30,41	37,47	0,00
	0,00	38,12	29,36	38,51	37,60	0,00	0,00	26,74	37,51	0,00
	0,00	38,03	29,36	39,25	37,59	0,00	0,00	30,38	37,11	0,00
	0,00	38,12	27,63	39,43	37,59	0,00	0,00	30,41	38,64	0,00
	0,00	38,11	24,78	39,26	36,68	0,00	0,00	30,41	37,47	0,00
	0,00	38,13	29,36	39,42	37,50	0,00	0,00	30,41	37,49	0,00
PtAT	0,00	0,00	23,01	30,52	0,00	0,00	0,00	22,22	30,79	0,00
	0,00	0,00	15,75	30,51	0,00	0,00	0,00	21,42	30,78	0,00
	0,00	0,00	19,68	28,72	0,00	0,00	0,00	15,03	30,80	0,00
	0,00	0,00	19,72	30,16	0,00	0,00	0,00	22,32	32,59	0,00
	0,00	0,00	19,71	30,51	0,00	0,00	0,00	19,52	29,89	0,00
	0,00	0,00	19,68	30,30	0,00	0,00	0,00	22,20	30,55	0,00
PtAT-R	0,00	0,00	14,49	29,14	37,14	0,00	0,00	17,17	34,70	38,39
	0,00	0,00	14,69	29,38	37,15	0,00	0,00	17,15	34,66	38,39
	0,00	0,00	14,49	29,38	37,07	0,00	0,00	17,16	34,67	38,39
	0,00	0,00	14,65	31,89	38,56	0,00	0,00	14,86	34,71	37,79
	0,00	0,00	9,16	27,00	37,21	0,00	0,00	14,52	32,74	36,42
	0,00	0,00	14,71	29,18	37,19	0,00	0,00	15,19	34,75	38,22

Gene	Cp				
	EB				
	35th	38th	40th	43rd	47th
PtRP49	31,00	31,00	31,00	31,00	31,00
	31,00	31,00	31,00	31,00	31,00
	31,00	31,00	31,00	31,00	31,00
	31,00	31,00	31,00	31,00	31,00
	31,00	31,00	31,00	31,00	31,00
	31,00	31,00	31,00	31,00	31,00
PtASTA	19,92	28,43	24,51	28,39	34,52
	18,52	27,49	21,04	28,67	34,46
	19,95	28,43	18,84	29,07	34,85
	19,92	27,49	24,57	28,39	34,36
	19,75	28,03	24,58	28,67	34,20
	19,64	28,43	24,58	29,07	34,46
PtASTA-R	32,69	37,33	38,02	38,37	38,64
	32,30	37,39	38,65	38,05	38,64
	33,87	37,83	37,40	38,46	39,13
	32,11	37,33	38,65	38,37	38,52
	33,03	37,37	38,66	38,05	38,33
	32,30	37,38	38,65	38,46	38,70
PtASTC	15,85	28,28	20,90	34,69	34,61
	15,84	23,16	19,77	34,07	34,77
	11,86	28,23	19,71	34,69	35,94
	15,83	28,26	21,71	34,61	34,60
	16,04	28,26	19,70	34,66	34,63
	15,84	28,28	21,60	34,64	34,59
PtASTC-R	36,50	36,98	37,60	36,98	36,98
	36,59	36,98	37,60	36,98	37,09
	34,74	37,83	35,77	37,83	37,83
	36,48	36,98	37,60	36,98	37,09
	36,52	36,98	37,61	36,73	36,90
	36,48	37,00	37,60	36,98	36,98
PtAT	31,64	32,88	30,82	32,54	33,24
	31,64	32,81	30,81	32,83	33,26
	31,55	30,79	30,77	32,52	33,26
	32,54	32,87	31,72	32,54	32,57
	31,58	32,83	30,83	32,81	33,26
	31,64	32,84	30,77	32,49	33,25
PtAT-R	34,82	35,35	32,52	37,31	37,94
	34,33	34,38	29,02	37,21	37,98
	34,76	35,29	32,44	36,50	36,81
	34,79	35,29	32,51	37,31	37,98
	34,80	35,55	32,53	37,21	37,96
	34,81	35,32	32,51	36,50	37,75

	Standard curves				
	**Copy number**	**Cp1**	**Cp2**	**Cp3**	**Average**
PtRP49	10^10	12,20	12,20	12,20	12,20
	10^9	15,33	15,34	15,33	15,33
	10^8	18,31	18,31	18,32	18,31
	10^7	21,90	21,91	21,91	21,91
	10^6	24,51	24,50	24,49	24,50
PtASTA	10^10	11,73	11,27	11,11	11,37
	10^9	13,21	13,22	13,30	13,24
	10^8	15,99	16,02	16,02	16,01
	10^7	18,87	18,76	18,87	18,83
	10^6	22,09	22,34	22,19	22,21
PtASTA-R	10^10	10,34	10,38	10,36	10,36
	10^9	13,22	13,19	13,21	13,21
	10^8	16,32	16,32	16,33	16,32
	10^7	19,42	19,38	19,38	19,39
	10^6	23,01	23,01	23,02	23,01
PtASTC	10^10	13,34	13,42	13,29	13,35
	10^9	15,23	15,23	15,24	15,23
	10^8	17,95	17,95	17,97	17,96
	10^7	20,33	20,35	20,34	20,34
	10^6	23,34	23,31	23,32	23,32
PtASTC-R	10^10	16,75	16,74	16,75	16,75
	10^9	18,97	18,94	18,99	18,97
	10^8	21,11	21,09	21,07	21,09
	10^7	23,77	23,84	23,81	23,81
	10^6	25,87	25,86	25,87	25,87
PtAT	10^10	14,63	14,64	14,64	14,64
	10^9	16,62	16,64	16,61	16,62
	10^8	19,79	19,8	19,79	19,79
	10^7	21,43	21,42	21,42	21,42
	10^6	23,58	23,55	23,56	23,56
PtAT-R	10^10	26,91	26,97	26,86	26,91
	10^9	28,02	28,09	27,98	28,03
	10^8	29,23	29,31	29,19	29,24
	10^7	30,69	30,73	30,71	30,71
	10^6	31,72	31,72	31,71	31,72
