# Supplementary material for: Expression profile of genes encoding allatoregulatory neuropeptides in females of the spider Parasteatoda tepidariorum (Araneae, Theridiidae)
Source: PLoS One. 2019 Sep 10;14(9):e0222274. doi: 10.1371/journal.pone.0222274 (PMC6736302; doi:10.1371/journal.pone.0222274)
Supplement: S1 Table — Allatostatin A, allatostatin B, allatostatin C and allatotropin and their receptor protein sequences of different insects species and accession number from the NCBI protein database used to search homologous proteins in P. tepidariorum. (PDF) [file pone.0222274.s015.pdf]

Neuropeptide	Order	Species	Accession number
Allatostatin A	Diptera	*Drosophila melanogaster*	NP_524489
			NP_001287511
	Hymenoptera	*Apis cerana*	PBC34787
	Lepidoptera	*Spodoptera frugiperda*	CAD32496
		Manduca sexta	AAB20830
	Hemiptera	*Plautia stali*	BAV78789
	Decapoda	*Neocaridina denticulata*	AIY69121
		Panulirus interruptus	BAF64528
	Cladocera	*Daphnia pulex*	EFX87432
	Ixodida	*Dermacentor variabilis*	ACC99603.1
			GI:186973145
Allatostatin A receptor	Lepidoptera	*Bombyx mori*	NP_001037035
		Spodoptera littoralis	ASO76367
	Diptera	*Ceratitis capitata*	XP_012159842
	Hemiptera	*D. obscura*	XP_022209096
	Cladocera	*D. pulex*	EFX71121.1
			GI:321460075
	Hymenoptera	*N. denticulata*	AIY69138
	Sessilia	*Megabalanus volcano*	ATQ64326
	Ixodida	*Ornithodoros moubata*	JAW06703
		Ixodes scapularis	EEC00437
		Rhipicephalus pulchellus	JAA56937
	Araneae	*Nephila clavipes*	PRD30488
	Mesostigmata	*Tropilaelaps mercedesae*	OQR73886
		Varroa jacobsoni	XP_022689235
Allatostatin B	Diptera	*D. melanogaster*	AHN57510
	Coleoptera	*Tribolium castaneum*	NP_001137202
			XP_001809338
		Leptinotarsa decemlineata	AIW62335
	Orthoptera	*Gryllus bimaculatus*	CAG28935
	Cladocera	*D. magna*	JAN91848
			JAL16932
	Decapoda	*Cherax quadricarinatus*	AWK57502
Allatostatin B receptor	Cladocera	*D. pulex*	EFX87704.1
			EFX84318
		D. magna	KZS13422
Allatostatin C	Diptera	*D. melanogaster*	NP_001162948
			NP_523542
	Coleoptera	*T. castaneum*	ACJ38500
	Hymenoptera	*Nasonia vitripennis*	ADM26612
		Ooceraea biroi	XP_011338324
		Odontomachus monticola	BBF97974
	Lepidoptera	*Helicoverpa armigera*	AXC25329
	Mesostigmata	*V. jacobsoni*	XP_022700594

		V. destructor	XP_022653394
	Decapoda	*Neocaridina denticulata*	AIY69122
	Cladocera	*D. pulex*	EFX85706
		D. magna	KZS21307
Allatostatin C receptor	Diptera	*D. melanogaster*	NP_649039
			AAN11677
	Hemiptera	*Rhodnius prolixus*	AHE41430
		Nilaparvata lugens	XP_022202031
		Myzus persicae	XP_022181644
	Lepidoptera	*Danaus plexippus*	OWR41004
	Phasmatodea	*Carausius morosus*	AOV81581
	Cladocera	*D. pulex*	EFX72686
	Mesostigmata	*V. jacobsoni*	XP_022700513
	Parasitiformes	*V. destructor*	XP_022653392
Allatotropin	Coleoptera	*T. castaneum*	NP_001137204
	Lepidoptera	*Helicoverpa armigera*	AAT92285
		M. sexta	AAB08759
	Diptera	*Aedes aegypti*	AAB06179
	Lepidoptera	*S. frugipeda*	CAD48594
		B. mori	XP_021207265
	Hymenoptera	*Bombus terrestris*	XP_003398476
	Collembola	*Orchesella cincta*	ODN02852
	Cladocera	*D. magna*	JAN79122
		D. pulex	EFX71302
	Ixodida	*I. scapularis*	EEC06620
Allatotropin receptor	Lepidoptera	*M. sexta*	ADX66344
		Helicoverpa armigera	AIT70966
		Chilo suppressalis	ALM88312
	Diptera	*A. aegypti*	AEN03789
		Anopheles darlingi	ETN65651
	Orthoptera	*Schistocerca gregaria*	AEX08666
			AKC92815
	Hemiptera	*Rhodnius prolixus*	AHE41431
	Hymenoptera	*B. terrestris*	NP_001291369
			XP_003402490
